# Arabidopsis VQ motif-containing proteins VQ1 and VQ10 interact with plastidial 1-deoxy-D-xylulose-5-phosphate synthase

**DOI:** 10.1038/s41598-024-70061-x

**Published:** 2024-08-15

**Authors:** Beatriz Gayubas, Mari-Cruz Castillo, José León

**Affiliations:** Instituto de Biología Molecular y Celular de Plantas (Consejo Superior de Investigaciones Científicas – Universidad Politécnica de Valencia), 46022 Valencia, Spain

**Keywords:** Arabidopsis, Chloroplasts, DXS, Isoprenoids, Photosynthesis, VQ proteins, Yeast two-hybrid, Biochemistry, Molecular biology, Plant sciences

## Abstract

VQ1 and VQ10 are largely unstructured homologous proteins with a significant potential for protein–protein interactions. Yeast two-hybrid (Y2H) analysis confirmed that both proteins interact not only with themselves and each other but also with other VQ and WRKY proteins. Screening an Arabidopsis Y2H library with VQ1 as bait identified 287 interacting proteins. Validation of the screening confirmed that interactions with VQ1 also occurred with VQ10, supporting their functional homology. Although VQ1 or VQ10 proteins do not localize in plastids, 47 VQ1-targets were found to be plastidial proteins. *In planta* interaction with the isoprenoid biosynthetic enzyme 1-deoxy-D-xylulose-5-phosphate synthase (DXS) was confirmed by co-immunoprecipitation. DXS oligomerizes through redox-regulated intermolecular disulfide bond formation, and the interaction with VQ1 or VQ10 do not involve their unique C residues. The VQ-DXS protein interaction did not alter plastid DXS localization or its oligomerization state. Although plants with enhanced or reduced *VQ1* and *VQ10* expression did not exhibit significantly altered levels of isoprenoids compared to wild-type plants, they did display significantly improved or diminished photosynthesis efficiency, respectively.

## Introduction

Extreme environmental stresses typical of a climate change scenario include factors related to water availability, such as drought and flooding, which have a significant impact on agriculture. In flooded areas, plants experience transient waterlogging or submersion until the water recedes. The effects of hypoxia during submergence and the subsequent reoxygenation play a crucial role in shaping plant growth and stress-related defense mechanisms^1,2^. Signaling pathways activated by oxygen deficiency are complex and primarily affect metabolism during respiration, but they also have a specific impact on mitochondrial and chloroplast function^3,4^. Hypoxic plants experience the simultaneous effects of limited O_2_ and enhanced production of NO and ROS. In chloroplasts, photosynthesis homeostasis is maintained by evolutionarily conserved thiol-based systems and antioxidative enzymes^5–7^. Oxygen fluctuations result in an altered redox balance and trigger nitroxidative stress during the onset of hypoxia and the subsequent re-oxygenation recovery^5,8^.

Proteins containing the FxxhVQxhTG motif, where x and h correspond to any amino acid and hydrophobic residues, respectively, belong to the VQ protein families, which are represented in all plants from bryophytes to flowering plants but not in ancestor chlorophytes^9^. Initially thought to be plant specific^10^, VQ proteins have also been identified in some fungi, lower animals, and bacteria, suggesting they have ancient origin and were evolutionary conserved^9^. Plant VQ protein families are encoded by multigene families with variable number of genes. In *Arabidopsis thaliana*, where the first VQ proteins were identified^11^, the VQ protein family comprises 34 members, with most of them possessing transcriptional regulatory activity^12^. Several VQ proteins interact physically and functionally with transcription factors of the WRKY family to control stress-related responses^13^ through a process depending on the integrity of the VQ motif and modulated by reversible phosphorylatio^14–16^. Plant VQ proteins are involved in regulating multiple developmental processes, including all transitions throughout the life cycle, as well as modulating plant responses to a wide range of abiotic and biotic stress factors^10,17^. Although the first identified VQ proteins SIGMA FACTOR-BINDING PROTEIN 1 (SIB1) and 2 (SIB2) are chloroplast-localized proteins^14,18^, the subcellular localization of most VQ proteins has not been experimentally addressed. However, predictions based on different algorithms point mostly to nuclear and cytoplasmic localization, with only a few seeming to be localized in chloroplasts and mitochondria^13,19,20^. Despite VQ proteins being involved in plant responses to multiple stress factors sharing the implication of oxidative stress processes, very little information is available on VQ proteins and their involvement in plant responses to oxidative stress. Five genes coding for Arabidopsis VQ1, VQ10, VQ24, VQ27, and VQ32 proteins have been identified as upregulated by hypoxia/reoxygenation, nitric oxide, and oxidative stress^21^, thus representing potential candidates for regulatory nodes in conditions related to limited oxygen availability in plants. Published data on these VQ proteins are restricted to a couple of articles describing the function of VQ10 in basal resistance to the necrotroph pathogen *Botrytis cinerea*^22^, and its role in regulating meristem growth, tolerance to oxidative stress, and sensitivity to nitric oxide^23^ as well as an article describing the function of VQ27 in NO-regulated mitochondrial biogenesis^24^. Here, we present data supporting that Arabidopsis VQ1 and VQ10 are largely unstructured homologous proteins with potential non-transcriptional regulatory functions primarily based on protein–protein interactions. VQ1 and VQ10 interacted with chloroplast proteins, thereby modulating photosynthesis and chloroplast function.

## Results

*Hypoxia-, NO-, and oxidative stress-upregulated genes VQ1 and VQ10 codes for paralogs with large potential in protein–protein interactions*.

**Figure 1:**
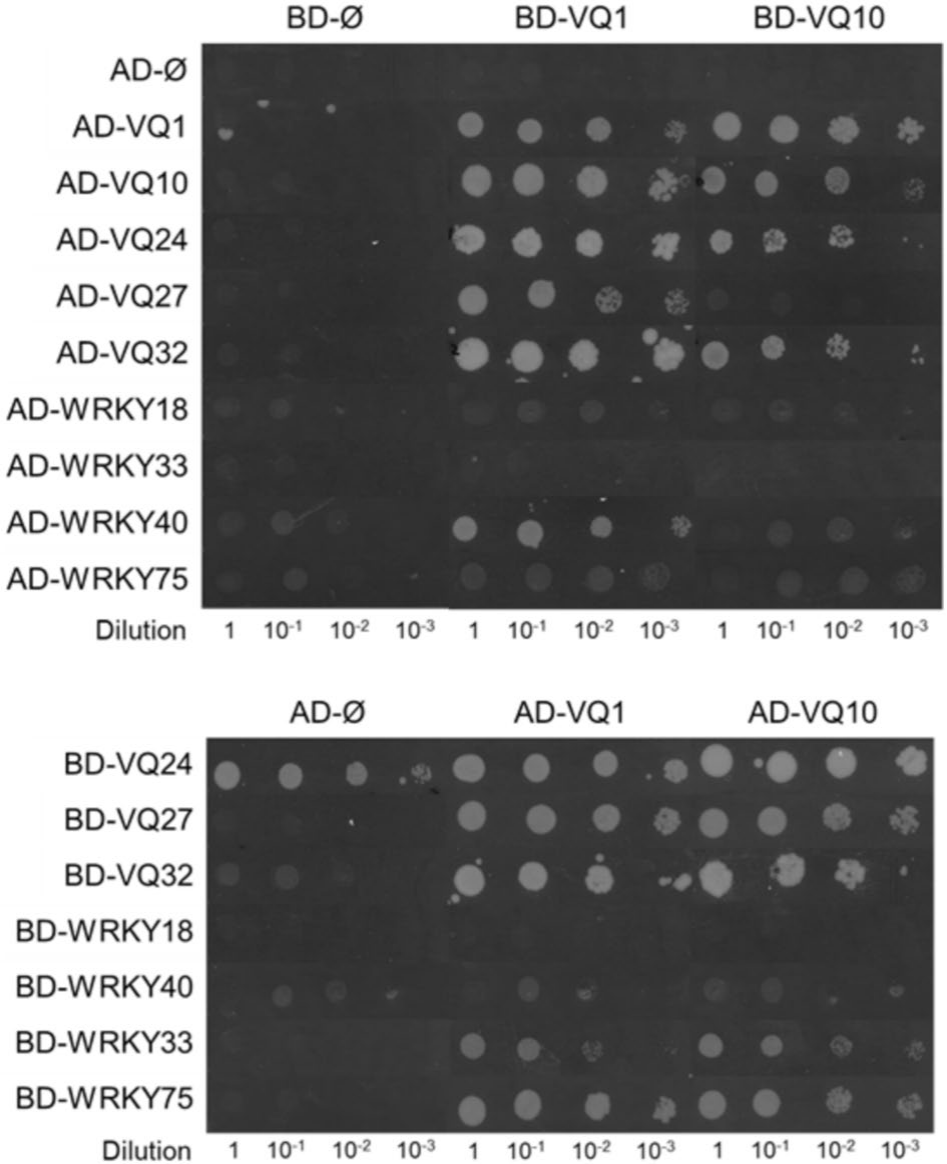
Yeast two-hybrid (Y2H) analysis of protein interactions of VQ1 and VQ10 proteins with other hypoxia-, NO-, and oxidative stress-inducible VQ and WRKY proteins. Interactions were evaluated using a Y2H mating system, with yeast cells transformed with the various proteins either fused to the GAL4 binding domain (BD) or activation domain (AD). Control experiments to verify no interaction were conducted using empty (Ø) BD or AD clones.

**Figure 2:**
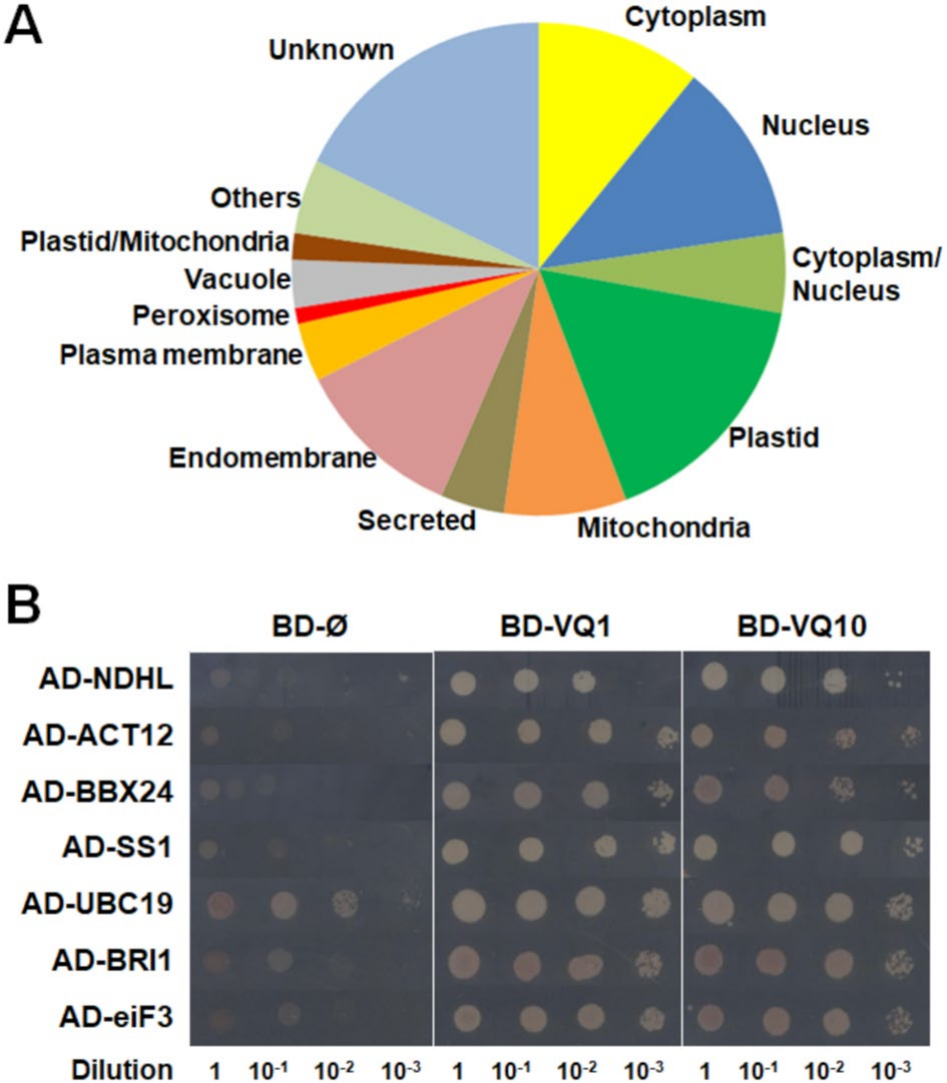
VQ1-interacting proteins identified in a Y2H screening of an Arabidopsis mating library. (**A**) Gene Ontology illustrates the subcellular localization of the identified interacting proteins. (**B**) Binary interactions with randomly selected clones were tested, confirming the interactions with VQ1 and VQ10.

Under natural or environmentally imposed limitation of oxygen availability, plants undergo the simultaneous effects of hypoxia and increased levels of NO and ROS^25^. We previously identified a set of genes coding for proteins containing the VQ motif that were co-regulated by these three factors^21^. These upregulated genes comprise five members of the VQ family (*VQ1*, *VQ10*, *VQ24*, *VQ27*, and *VQ32*) and four members of the WRKY transcription factor family (*WRKY18*, *WRKY33*, *WRKY40*, and *WRKY75*). All four WRKY proteins have been reported to act as transcription factors^26–29^, but only VQ24 and VQ32 had been predicted to be localized in nucleus and to function as transcriptional regulators^12^. In turn, all these VQ and WRKY proteins have been reported to interact with other proteins, and two of them, VQ1 and VQ10, might bind RNA according to in silico predictions (Supplementary Table S1). Interestingly, the three VQ proteins with no reported transcriptional regulatory function were predicted, though not experimentally assessed yet, to be localized in cellular compartments other than nucleus, mainly the cytoplasm, chloroplasts, or cell membrane (Supplementary Table S1), which may reflect other regulatory functions not related to transcription. The differential specific features displayed by VQ1 and VQ10 (Supplementary Table S1), together with the large degree of amino acid identity and conservation in their sequences, and the predicted 3D structural models, prompted us to functionally characterize VQ1 and VQ10 proteins. Since the interaction of WRKY33 with VQ proteins was previously reported^13^, we investigated the interactions between all members of the subset of hypoxia-, NO-, and oxidative inducible VQ and WRKY proteins described above. Using yeast two-hybrid (Y2H) assays with VQ1 and VQ10 as baits, we confirmed that VQ1 and VQ10 interacted with themselves, each other, and with VQ24, VQ27 and VQ32 proteins as well as with WRKY18, WRKY33, WRKY40 and WRKY75 transcription factors (Fig. 1). The interactions of VQ1 and VQ10 with WRKY33 and the interaction of VQ10 with VQ27 were only detected when VQ1 and VQ10 were fused to the GAL4 activation domain and VQ27 or WRKY33 to the GAL4 DNA binding domain, and the interaction of VQ1 and VQ10 with WRKY18 was very weak (Fig. 1). Additionally, we observed that WRKY40 and VQ24 fused to the GAL4 DNA-binding domain were able to partially or fully transactivate, respectively (Fig. 1). These data suggest that VQ1 and VQ10 have the potential to homodimerize and heterodimerize among themselves and with other VQ proteins, but also that either single VQ proteins or complexes might interact with WRKY proteins, perhaps modulating their transcriptional activity. To test whether the interaction of VQ proteins was specific for just other VQ proteins or WRKY transcription factors, a Y2H screening of a universal normalized Arabidopsis Mate&Plate library was performed using VQ1 as bait. The screening yielded 504 prey positive clones that, after sequencing, corresponded to 287 unique different interacting proteins (Supplementary Table S2). Among them, 47 were plastidic, 34 nuclear, 31 cytoplasmic, 28 mitochondrial, and 23 endomembrane-associated proteins, and the rest have either several localizations or remain unknown (Supplementary Table S2, Fig. 2A). Despite the binary interactions between VQ1 or VQ10 and WRKY18, WRLY33, WRKY40 and WRKY75 were detected though with different affinities (Fig. 1), none of these transcription factors were identified as proteins interacting with VQ1 in the Y2H screening. The only member of the WRKY family identified in the Y2H screening was WRKY12 (Supplementary Table S2). We cannot rule out the possibility that despite sequencing more than 500 clones, we are still far from analyzing the whole library thus reducing the probability of identifying the less represented transcripts. To test the reliability of the screening, we confirmed the interactions of several randomly selected prey clones with different subcellular localizations by testing binary interactions in the yeast mating system with the VQ1 bait. Every analyzed interaction was not only confirmed for VQ1 but also detected when VQ10 was used as bait (Fig. 2B), suggesting that both proteins are likely homologs in terms of protein–protein interactions. Gene ontology (GO) analysis allowed the identification of a significant over-representation of biological processes related to photosynthesis-driven primary metabolism of carbon, nitrogen, and sulfur including sugars and organic acids, as well as N- and S-containing amino acids (Supplementary Table S3). The significant over-representation of chloroplast-related functional categories among the identified VQ1-interacting proteins suggests that VQ1, and likely VQ10 too, might play relevant roles on regulating chloroplast function. Among the 47 plastid proteins interacting with VQ1, 28 proteins were enzymes, 14 were essential for photosystems I and II assembly, maintenance, or protection, and four were transporters of metabolites or proteins into the chloroplasts (Supplementary Table S4).

### VQ1 or VQ10 interact in planta with plastid DXS protein

**Figure 3:**
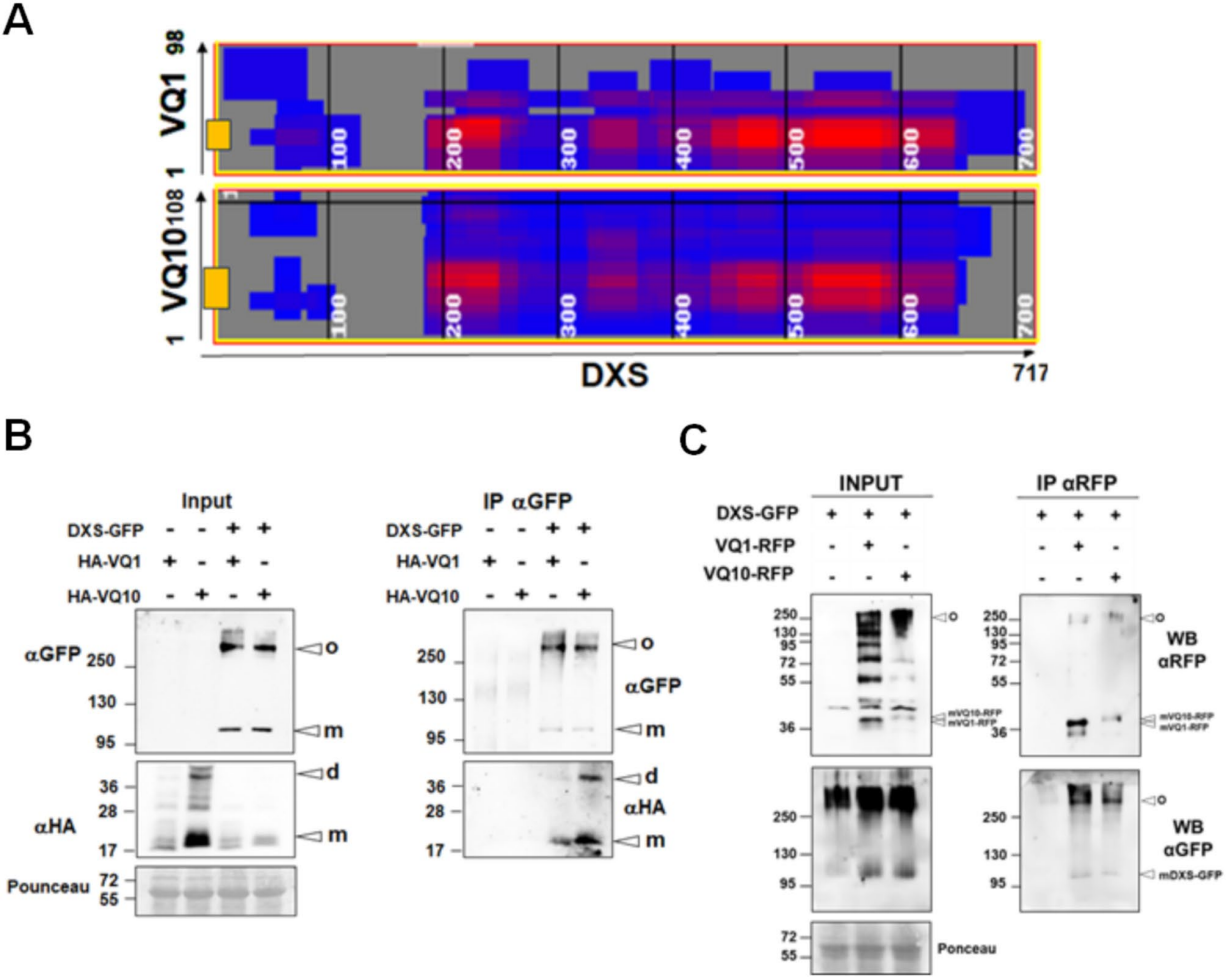
Co-immunoprecipitation of HA-VQ or VQ-RFP proteins and DXS-GFP proteins. (**A**) In silico prediction of the interaction between DXS and either VQ1 or VQ10 using iFrag algorithm. VQ motifs of VQ1 and VQ10 are represented by yellow boxes. *Nicotiana benthamiana* leaves from plants were infiltrated with Agrobacterium transformed with (**B**) *35S:3xHA-VQ1* (HA-VQ1) or *35S:3xHA-VQ10* (HA-VQ10) and *35S:DXS-GFP* (DXS-GFP) and (**C**) *35S:VQ1-RFP(VQ1-RFP)* or *35S:VQ10-RFP* (VQ10-RFP) and *35S:DXS-GFP* (DXS-GFP). By three days after agroinfiltration they were used to make total protein extracts (Input) and to immunoprecipitate (IP) with magnetic beads coated with a-GFP antibodies (**B**) or magnetic beads coated with a-RFP antibodies (**C**). Protein extracts were prepared in the presence of 0.5 mM DTT. A high molecular mass oligomer (o) as well as the DXS monomer (m) were detected. Proteins were detected by Western blot with the indicated antibodies. Equal loading was checked in by staining membrane with Ponceau S. The position of molecular mass markers (kDa) is indicated at the left side of each panel.

**Figure 4:**
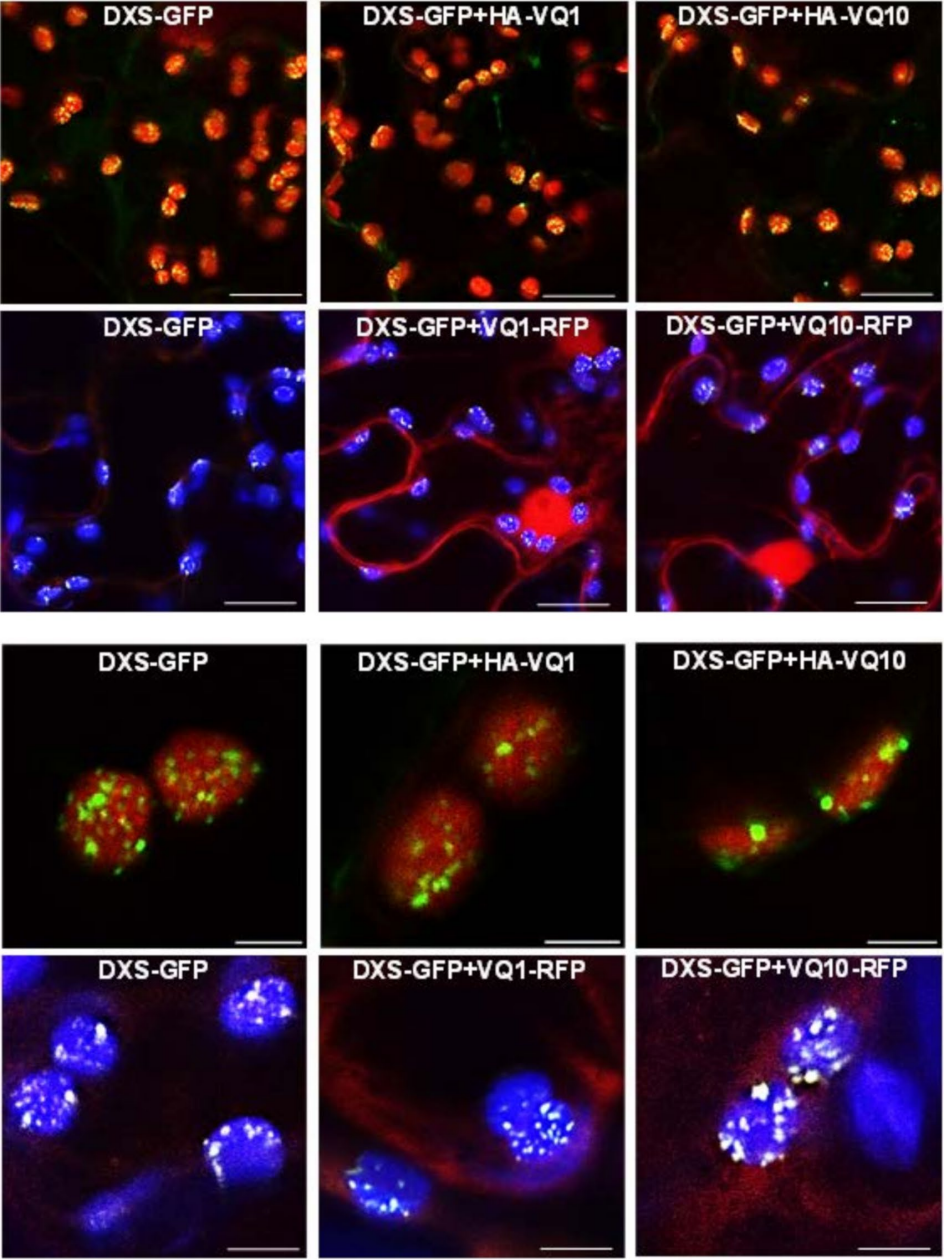
Subcellular localization patterns of DXS, VQ1 and VQ10 upon co-expression. *Nicotiana benthamiana* leaves were infiltrated with Agrobacterium transformed with *35S:3xHA-VQ1* (HA-VQ1), *35S:3xHA-VQ10* (HA-VQ10), *35S:VQ1-RFP* (VQ1-RFP), *35S:VQ10-RFP* (VQ10-RFP), or *35S:DXS-GFP* (DXS-GFP) as indicated. Confocal microscopy images were taken by 3 days after agroinfiltration. Merging green for GFP and red for chloroplast autofluorescence (panels in first and third rows), and green for GFP, red for RFP, and blue for chloroplast autofluorescence (panels in second and fourth rows) are shown. Bar size in panels of rows 1–2 and 3–4 corresponded to 3 and 15 µm, respectively.

To assess whether the chloroplast-localized VQ1-interacting proteins detected in the yeast system also interact *in planta* with VQ1 and VQ10, we selected DXS (1-deoxy-D-xylulose-5-phosphate synthase) because of its relevance as key enzyme in the 2-C-methylerythritol 4-phosphate (MEP) pathway controlling isoprenoid biosynthesis in plastids^30^. Prediction of protein–protein interactions using the iFrag algorithm between DXS and either VQ1 or VQ10 suggested that both VQ proteins could potentially interact through their respective VQ domain and flanking regions with three different domains of the DXS protein (Fig. 3A). We tested the potential interaction *in planta* by co-immunoprecipitation (CoIP) in *Nicotiana benthamiana* leaves that were agroinfiltrated with constructs expressing VQ1 or VQ10 tagged with 3xHA epitopes in the N-terminus (HA-VQ1 and HA-VQ10) and DXS-GFP. Figure 3B shows that anti-GFP antibodies allowed the precipitation of DXS-GFP and the co-immunoprecipitation of the HA-tagged VQ1 or VQ10 proteins. Two forms of DXS-GFP were detected, high molecular mass DXS-GFP oligomers of more than 250 kDa and 102 kDa corresponding to the DXS-GFP monomer, both in the input and the IP (Fig. 3B). We also found that both dimers and monomers of VQ1 and VQ10 were co-immunoprecipitated (Fig. 3B). We checked that the interaction between VQ proteins and DXS was not dependent on the position of the tag in VQ10 proteins because co-agroinfiltration of Nicotiana with VQ1-RFP or VQ10-RFP and DXS-GFP followed by IP with anti-RFP magnetic beads resulted also in the co-immunoprecipitation of both monomer and oligomers of DXS (Fig. 3C). We also checked whether the *in planta* interaction between DXS and either VQ1 or VQ10 altered the DXS localization by confocal microscopy analysis of *Nicotiana benthamiana* leaves transiently co-expressing DXS-GFP and VQ1-RFP or VQ10-RFP. Figure 4 shows that no changes in the spotted localization of DXS in chloroplasts were detected when VQ1 or VQ10, tagged with either HA or RFP, were co-expressed with DXS.

### Oligomer-monomer distribution of DXS is redox sensitive

**Figure 5:**
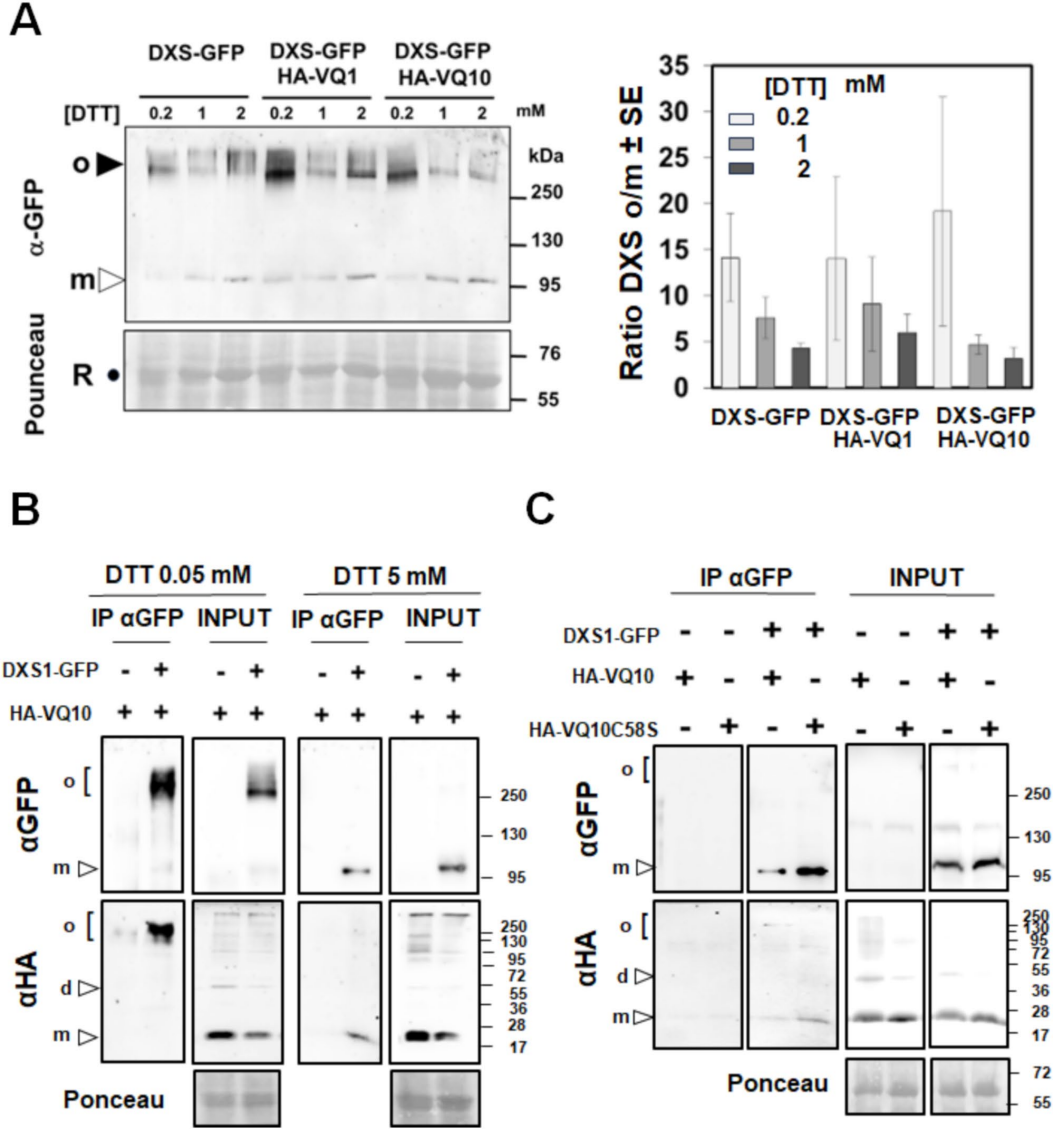
VQ1 and VQ10 proteins altered DXS aggregation and oligomerization patterns in chloroplasts. (**A**) *Nicotiana benthamiana* plants were transiently transformed by agroinfiltration with plasmids harboring *35S:DXS-GFP* (DXS), *35S:3xHA-VQ1* (HA-VQ1), and *35S:3xHA-VQ10* (HA-VQ10) as indicated plus *p19* plasmid to avoid posttranscriptional silencing. Three days after agroinfiltration, total protein extracts were prepared from leaves co-transformed with the indicated combination of plasmids and analyzed by Western blot with a-GFP antibody to detect DXS. The GFP signals from the bands corresponding to oligomers (o, solid triangle) and monomers (m, clear triangle) are shown in plants under the increasing DTT concentrations indicated. The bottom panel shows de Ponceau-stained Rubisco (R, solid dot) to assess for equal loading. The right panel shows the quantification by ImageQuant TL software as the mean of the oligomer/monomer ratio ± standard error (SE) of four independent biological replicates for each condition. (**B**) CoIP of DXS and VQ10 under low reducing (0.05 mM DTT) and strong reducing (5 mM DTT) conditions. IP was performed with anti-GFP magnetic beads and the levels of DXS-GFP and HA-VQ10 analyzed by western blot with the indicated antibodies. The positions of DXS monomer (m) and oligomers (o) in the upper panels and VQ10 monomer (m), dimer (d), and oligomers (o) in the lower panels are marked at the right side. Molecular mass markers in kDa are shown at the left side of panels. (**C**) CoIP of DXS-GFP and either wild type HA-VQ10 or mutated HA-VQ10(C58S) proteins was performed at 5 mM DTT.

**Figure 6:**
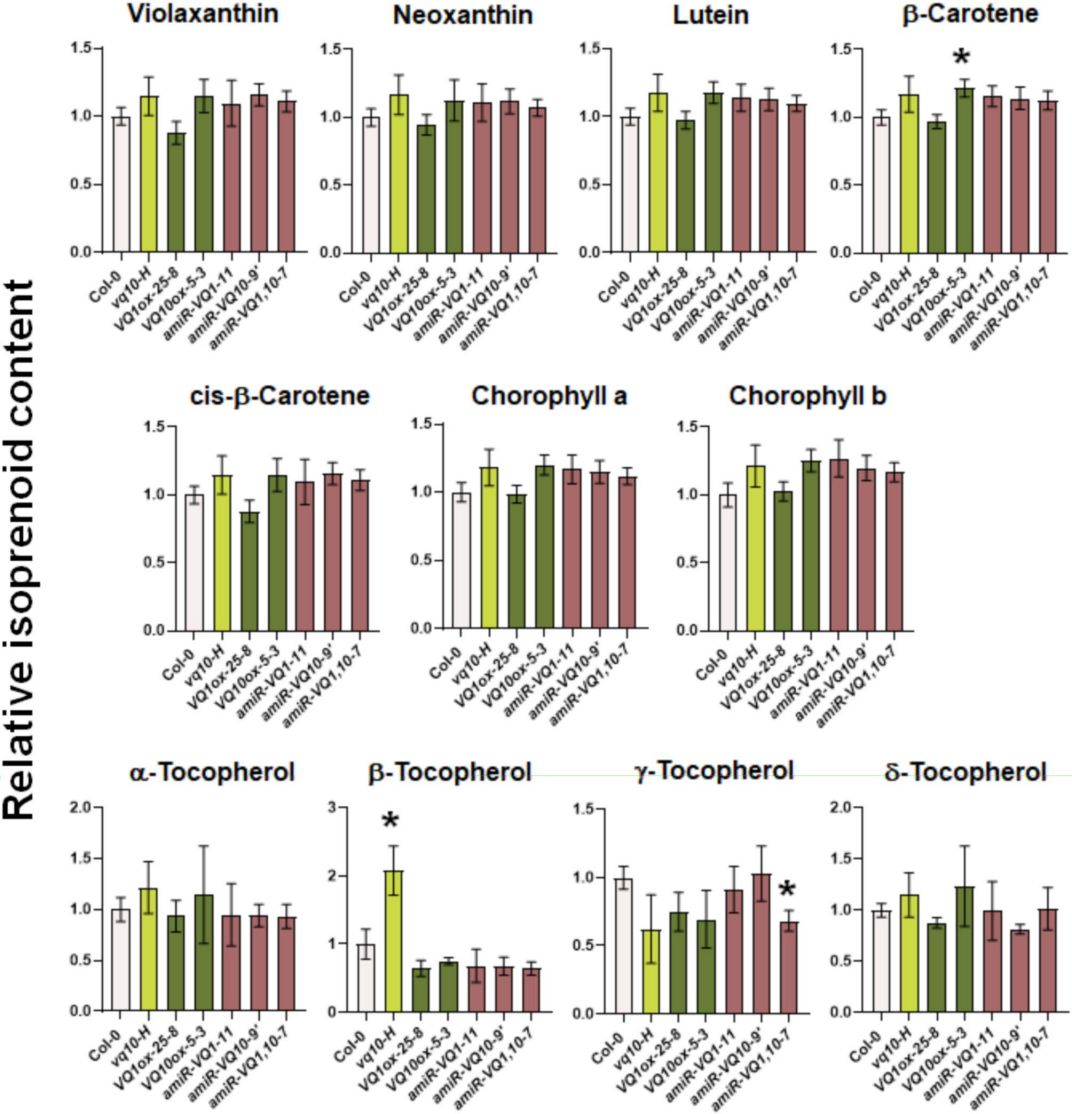
Quantification of isoprenoid metabolites in seedlings with altered expression of VQ1 and VQ10 genes. Values represented the mean of three biological replicates ± SEM and were made all relative to the content in wild type plants. Clear bars corresponded to wild type Col-0 plants, light green to the hypermorphic *vq10-H* mutant, dark green to overexpressing transgenic lines, and brown to downexpressing amiRNA lines. Statistical significance was analyzed by student’s *t*-test comparing each genotype to Col-0. *p < 0.05.

It has been recently reported that DXS is active as a dimer, and its function is under the control of a dimerization/monomerization mechanism that is also connected to aggregation processes, presumably due to oligomerization. This regulatory mechanism depends on the availability of substrates of the methylerythritol 4-phosphate (MEP) pathway that can allosterically bind to DXS exerting feed-back inhibition^31^. We have found that the oligomerization state of DXS is sensitive to thiol-dependent redox regulation, as an increased monomer/oligomer ratio was detected when reducing power supplied by dithiothreitol (DTT) was increased (Fig. 5A). To analyze whether the VQ-DXS interactions altered the aggregation state of DXS-GFP or its sensitivity to redox regulation, we tested whether the co-expression of VQ1 or VQ10 together with DXS altered its oligomerization. Figure 5A shows the levels of DXS monomer and oligomer under increasing reductive conditions achieved by treatment with DTT. Neither the oligomer/monomer ratio of DXS nor the sensitivity to DTT in promoting monomerization was significantly altered when VQ1 or VQ10 were co-expressed with DXS (Fig. 5A). We also confirmed that VQ10 was co-immunoprecipitated with DXS when CoIP was performed either under mild or strong reducing conditions. Figure 5B shows that the CoIP of VQ10 was detected regardless of the redox conditions, though under low reducing conditions, the IP of DXS oligomers led to the CoIP of mainly VQ10 oligomers whereas under increased reducing conditions, the IP of mostly DXS monomer correlated with the CoIP of VQ10 monomer. These data suggest that the *in planta* interaction between DXS and VQ10 did not rely solely on intermolecular disulfide bond formation but occurred through a redox-independent interaction likely mediated by another motif, which could well be the VQ motif as predicted in silico (Fig. 3A). We have confirmed that DXS-VQ10 interaction occurs fully independent of the C residue because after co-expression of DXS-GFP and a mutated version of VQ10 replacing C58 by S (HA-VQ10C58S), the IP of DXS-GFP also led to CoIP of HA-VQ10C58S protein (Fig. 5C). The subcellular localization of the mutated VQ10 version was not altered either, as shown by confocal microscopy analysis in Nicotiana leaf cells expressing VQ10(C58S)-RFP (Supplementary Fig. S1). Since no significant effect on the oligomerization state of DXS by VQ1 or VQ10 was detected, we wonder whether DXS-VQ interactions could affect DXS function. DXS is a key enzyme in the MEP pathway of carotenoids and tocopherols biosynthesis, so we checked whether overexpressing or repressing *VQ1* or *VQ10* gene expression in transgenic plants led to altered levels of isoprenoid metabolites. We used the previously reported hypermorphic *vq10-H* mutant and transgenic lines overexpressing the full cDNAs coding for 3xHA-VQ1 or 3xHA-VQ10 proteins^23^, as well as plants with reduced *VQ1* and/or *VQ10* gene expression due to the expression of artificial microRNAs (amiRNA) specifically targeting either or both together. By FPLC followed by photodiode array or fluorescence detection in whole seedling extracts of the different transgenic genotypes and wild-type plants, we quantified the levels of carotenoids violaxanthin, neoxanthin, lutein, β-carotene, and cis-β-carotene; the chlorophylls a and b; and the tocopherols α, β, δ, and γ. Values summarized in Fig. 6 point to no significant alterations in any of the isoprenoid metabolites tested with the only exception of the twofold increase in β-tocopherol levels in *vq10-H* mutant compared to wild type Col-0 plants, thus suggesting either DXS-VQ interaction did not affect to DXS activity or that some other step downstream in the pathway is the rate-limiting step in this biosynthesis pathway.

### Effect of changes in VQ1 and VQ10 expression on photosynthesis and greening during de-etiolation

**Figure 7:**
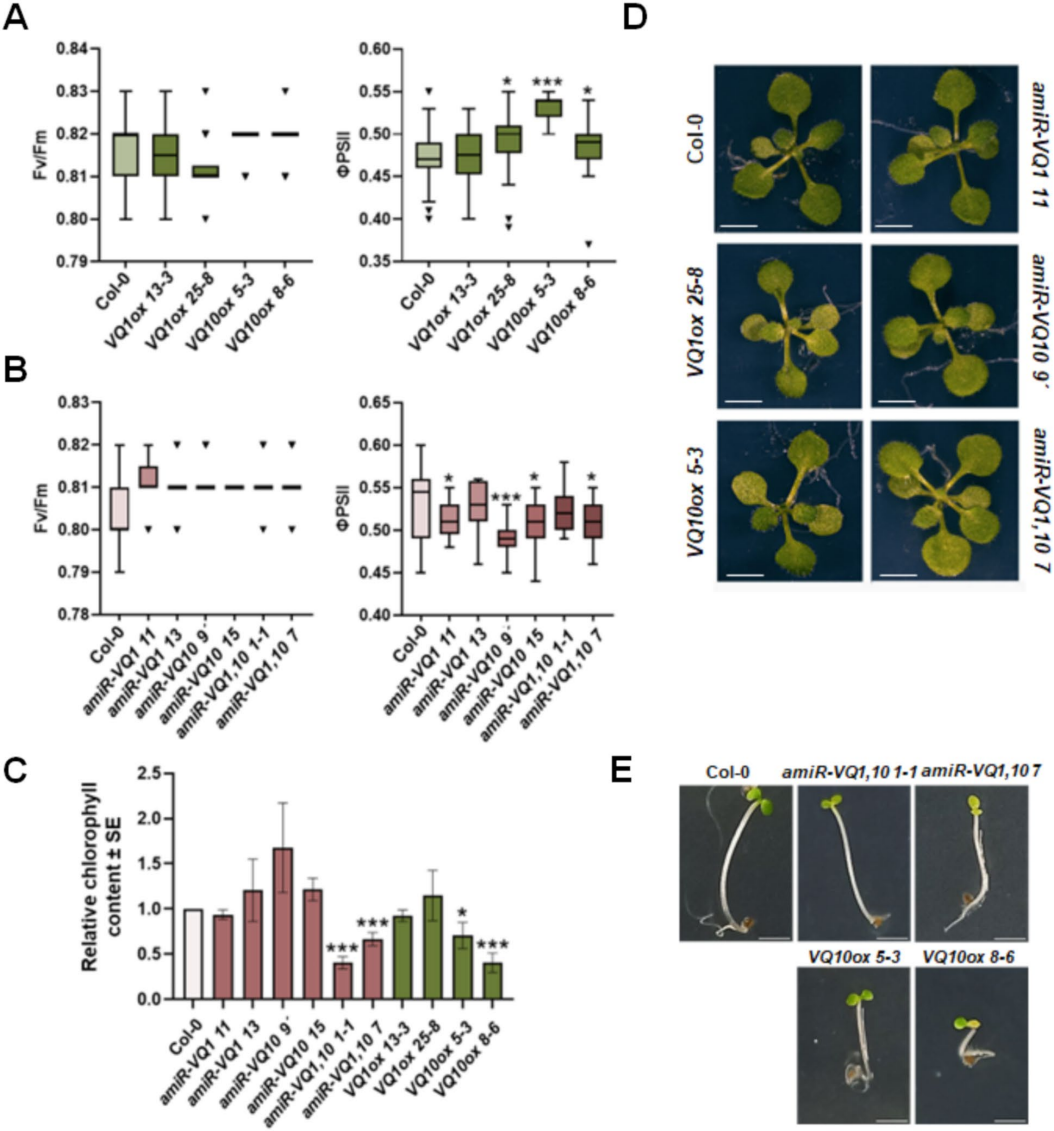
Photosynthetic activity and de-etiolation of plants with enhanced or reduced *VQ1* and *VQ10* gene expression. The maximum quantum efficiency (Fv/Fm) and effective quantum yield of PSII reaction centers (ΦPSII) of (**A**) overexpressing plants or (**B**) expressing amiRs (between 10 and 15 replicates) of the indicated genotypes was measured after incubation of plants under darkness for 20 min before being exposed to a flash of actinic light (2000 µmol m^−2^ s^−1^). Box plots are shown displaying the median (horizontal bar inside the box), and the error interval. (**C**) De-etiolation process was assessed by measuring the total chlorophyll content in seedlings (three replicates) of the indicated genotypes. Seed germination was activated by a 6 h light exposure, then seedlings grew skotomorphogenically under darkness for 3 days, and finally were re-exposed to light for 24 h before chlorophyll quantification. (**D**) Representative images of rosettes from the indicated genotypes. (**E**) Representative images of etiolated seedlings after de-etiolation for Col-0 and the genotypes with significant reduction in chlorophyll synthesis. White bars in panels (**D**) and (**E**) represent 2 mm. Statistical significance was assessed by unpaired *t*-test comparing each genotype to Col-0. *p < 0.05; **p < 0.005; and ***p < 0.001.

The interaction of VQ1 and VQ10 proteins with plastid proteins, some of which are involved in the assembling, maintenance, or protection of photosystems I and II, suggest that these VQ proteins could play a role in modulating plant photosynthesis. The transgenic genotypes with either enhanced or decreased expression of *VQ1* and *VQ10* genes, along with Col-0 background plants, were analyzed for maximum and efficient photosynthetic activity. Figure 7A shows that genotypes with enhanced expression of *VQ1* or *VQ10* genes showed significantly higher effective quantum yield of PSII reaction centers (ΦPSII) than wild type Col-0 plants, with no significant changes in the maximum quantum efficiency (Fv/Fm) of PSII. In turn, among amiRNA lines, *amiR-VQ1 11*, *amiR-VQ10 9´,* and *amiR-VQ1,10 10*-*7* displayed significantly lower ΦPSII than Col-0 plants, with no changes in the maximum quantum efficiency of PSII (Fig. 7B). No visible phenotypic alterations were detected in plants with either enhanced or reduced expression of *VQ1* or *VQ10* genes (Fig. 7D). Besides Fv/Fm and ΦPSII, the non-photochemical quenching parameters (NPQ and qN), and the proportion of open PSII (qP) were measured in plants with reduced or enhanced *VQ1* or *VQ10* expression. Minor effects were observed in NPQ and qN parameters, but increased qP were observed in overexpressing plants and in some of the amiR plants (Supplementary Fig. S2), suggesting that the proportion of open PSII centres is higher in plants with altered *VQ* expression likely due to low saturation of photosynthesis by light. We also tested whether the effects of altered *VQ1* or *VQ10* expression on chloroplast function could be related to a potential involvement of VQ proteins in the chloroplast biogenesis during de-etiolation. We found that chlorophyll biosynthesis during greening was partially impaired either in the best double amiRNA lines (*amiVQ1,10 1*-*1* and *amiVQ1,10 7*) or in the *VQ10* overexpressing (*VQ10ox 5*-*3* and *VQ10ox 8*-*6*) plants (Fig. 7C). *amiR-VQ1* and *amiR-VQ10* plants with reduced chlorophyll displayed paler green cotyledons than Col-0 with no alteration in hypocotyl length, while overexpressing plants had also paler cotyledons and shorter hypocotyls (Fig. 7E). The fact that we found a similar phenotype of deficient greening in plants with augmented or reduced *VQ10* transcript levels suggest that either tagged VQ proteins are not as active as the endogenous proteins and they eventually affected negatively to them by scavenging based on protein interaction, or that the effect on the de-etiolation process is triggered when *VQ1* or *VQ10* expression is outside of a relatively narrow threshold. Together, these findings suggest that VQ1 and VQ10 could regulate chloroplast processes, including photosynthetic activity and de-etiolation, through uncoupled complex mechanisms yet to be more deeply explored.

**Figure 8:**
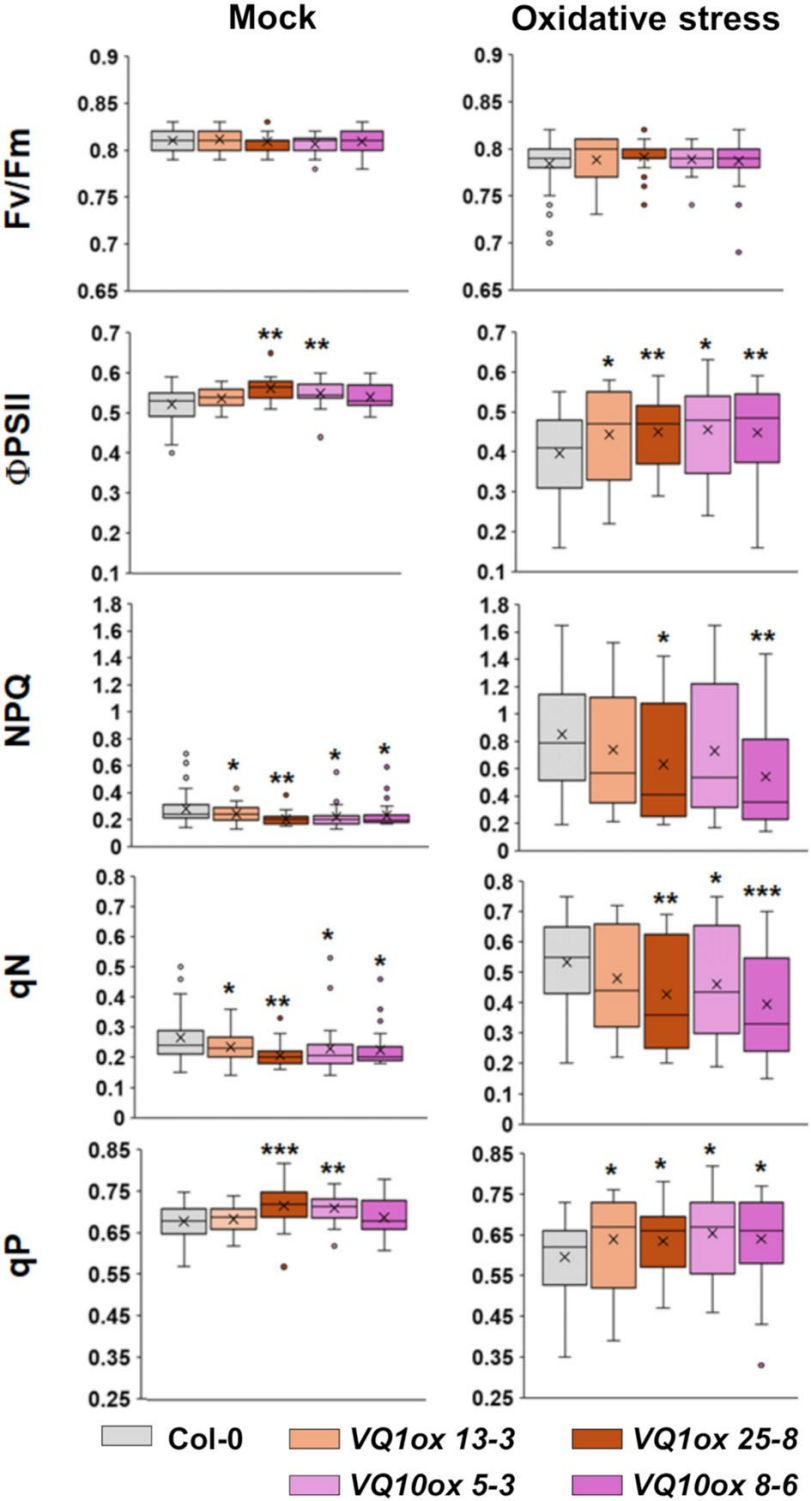
Measurement of photosynthetic parameters in plants with enhanced*VQ1* or *VQ10* gene expression. The maximum quantum efficiency (Fv/Fm), effective quantum yield of PSII reaction centers (ΦPSII ), non-photochemical quenching (NPQ) and its related qN, and the proportion of open PSII centres (qP) were measured in untreated (mock) plants as well as in plants treated with 30 µM methyl viologen (MV) for 16 h (oxidative stress) of the indicated genotypes. Box plots are shown displaying the median (horizontal bar inside the box), and the error interval. Statistical significance was assessed by unpaired *t*-test comparing each genotype to Col-0 under mock or stress conditions. *p < 0.05; **p < 0.005; and ***p < 0.001.

Since VQ1 and VQ10 levels are low under non-stressed conditions but they are upregulated by factors such as oxidative stress^21^, we have checked whether VQ1 and VQ10 proteins could modulate the efficiency of photosynthesis in plants undergoing oxidative stress after treatment with methyl viologen (MV). We measured Fv/Fm, ΦPSII, NPQ, qN, and qP parameters in wild type Col-0 or different VQ1 or VQ10 overexpressing plants either mock treated or treated with 30 µM MV. The oxidative stress triggered no significant changes in Fv/Fm in any of the tested genotypes compared to unstressed conditions, but lowered ΦPSII in all of them (Fig. 8). However, the oxidative stress-triggered reduction in the effective quantum yield of PSII was significantly ameliorated in plants overexpressing *VQ1* or*VQ10* genes (Fig. 8). On the other hand, treatment with MV largely increased the values of NPQ and qN and slightly decreased the values of qP in all genotypes, but again this enhancement of NPQ and qN as well as the reduction in qP was significantly counteracted in plants with higher VQ1 or VQ10 expression (Fig. 8). These findings together suggest that VQ1 and VQ10 may contribute to enhanced photosynthetic efficiency by ameliorating heat dissipation and closure of reaction centres.

## Discussion

When plants face hypoxic conditions either throughout development in metabolically active tissues^32,33^ or due to environmental stress^34^, they undergo the effect of increased levels of endogenous NO and ROS^35–37^ accompanying the limited availability of oxygen. In a search for genes co-regulated by NO, hypoxia, and oxidative stress, we found a set of five upregulated genes coding for proteins containing the VQ motif^21^, which comprises VQ1, VQ10, VQ24, VQ27, and VQ32. *VQ1* and *VQ10* genes code for small proteins with a high degree of homology in their amino acid sequence and a large proportion of disordered sequences. These two proteins, unlike most of the Arabidopsis VQ protein family, do not seem to bind DNA and regulate transcription^12^ (Supplementary Table S1). Therefore, their regulatory functions are likely exerted through other biomolecular interactions. Regarding this, the regulatory mode of action of many VQ proteins has been linked to the physical interaction with transcription factors of the WRKY family^13^. Here, we demonstrated that VQ1 and VQ10 proteins interact with each other and with themselves, as well as with the other three VQ proteins and four WRKY proteins induced by hypoxia, NO, and oxidative stress (Fig. 1). These multiple interactions suggest that VQ and WRKY proteins likely form different regulatory complexes, thus opening the possibility to regulate a wide variety of processes in a combinatorial way, sometimes through WRKY-mediated modulation of transcription. The formation of high-order regulatory complexes either through homodimerization or heterodimerization, as well as oligomerization to different degrees, is thus likely, but more work will be required to elucidate the nature and composition of these regulatory complexes as well as their mode of action in different processes. Despite the potential relevance of the interactions between VQ1 or VQ10 with WRKY transcription factors, we presented here evidence suggesting that VQ1 and VQ10 are promiscuous in their interaction with a wide array of functionally diverse proteins with different subcellular localizations. A Y2H screening of a universal Arabidopsis normalized library using VQ1 as bait allowed us to identify close to three hundred proteins (Supplementary Table S2). Among VQ1-interacting proteins, we found proteins with all different subcellular localizations (Fig. 2A), and those that were tested with VQ10 as bait also displayed interaction (Fig. 2B), thus supporting the functional homology between VQ1 and VQ10 at the protein–protein interaction level. Cytoplasmic, nuclear, and endomembrane-associated VQ1-interacting proteins identified in the screen are compatible with the subcellular localization of VQ1 and VQ10, which are localized in these compartments. However, we also found 47 VQ1-interacting proteins with plastid localization (Supplementary Table S4), and because our data suggested no plastid localization for either VQ1 or VQ10, these potential interactions should occur in the transit of target proteins from the cytoplasm to the organelle. We have confirmed the *in planta* interaction of VQ1 or VQ10 with one of the plastidial proteins identified in the Y2H screening, 1-deoxy-D-xylulose-5-phosphate synthase (DXS). VQ and DXS proteins were co-immunoprecipitated from plants transformed with DXS-GFP and HA-VQ proteins (Fig. 3B). The co-immunoprecipitation approach allowed identifying two forms of DXS protein, monomers of 102 kDa corresponding to DXS-GFP protein and oligomers of molecular mass above 250 kDa (Fig. 3B). It has been previously reported that the balance between DXS monomers and oligomers is regulated by products of the methylerythritol 4-phosphate (MEP) pathway through an allosteric mechanism^31^. Our data showed that the co-expression of VQ1 or VQ10 and DXS did not affect either the oligomerization of DXS or the DTT-promoted monomerization (Fig. 5A). Moreover, redox conditions did not affect the interaction between DXS and VQ proteins (Fig. 5B), suggesting that the DXS-VQ protein interaction did not depend on the formation of intermolecular disulfide bonds involving the unique C residues of VQ1 or VQ10 proteins. This was also confirmed by the co-immunoprecipitation of a mutated version of VQ10, with the unique C residue mutated to S, together with the immunoprecipitated DXS (Fig. 5C). The in silico models we have used to predict protein–protein interactions between DXS and VQ1 or VQ10 suggested that the interaction likely occurs through the corresponding VQ motifs (Fig. 3A). The VQ motif has been previously reported to be essential for VQ proteins interaction with WRKY transcription factors^14^.

Alternatively, VQ1 or VQ10 could modulate DXS oligomerization through a redox regulator intermediate. Among the VQ1-interacting proteins identified in this work (Supplementary Table S2), the plastidial Ferredoxin-NADP reductase isozyme 2 (FNR2) maintains the supply of reduced ferredoxin under non-photosynthetic conditions in roots, affecting plastid function and nitrogen assimilation^38^. Noteworthy, among the VQ1-interacting proteins, we also found several enzymes of the N assimilation pathway, including cytosolic and plastid glutamine synthetases GS1 and GS2, a 2-oxoglutarate and Fe(II)-dependent oxygenase, and the plastid-localized Fd-GOGAT (Supplementary Table S2), all key enzymes in the GS-GOGAT cycle catalyzing the incorporation of reduced N in form of ammonia into carbon skeletons. Together, these findings suggest that VQ1 and VQ10 could be important regulators of N-related photosynthetic metabolism. Another VQ1-interacting protein AT5G65840, an uncharacterized member of the thioredoxin multigene family, was also identified in this work, and it could be a more likely intermediate for the redox modulation of DXS monomerization exerted by VQ proteins.

Despite VQ1 and VQ10 interacted with DXS, neither the oligomerization state of DXS, nor the subcellular localization pattern of DXS were altered upon co-expression of *VQ1* and *VQ10* (Fig. 4). Moreover, DXS function was not altered either in plants with enhanced or repressed *VQ1* and *VQ10* expression, as demonstrated by the absence of significant changes in the endogenous levels of DXS-derived isoprenoids (Fig. 6). Since we have not found plastidic localization for VQ1 or VQ10 proteins, the interaction with plastid proteins should occur outside the chloroplast during the transit in the cytoplasm to the organelle. The interaction between VQ1 or VQ10 with DXS, and potentially with other plastidic proteins identified in the screening, could either retain these target proteins in the cytoplasm, thus scavenging them from their regular destination, or conversely, facilitate the transit of these proteins to the organelle. It is tempting to speculate that at low levels of *VQ1* or *VQ10* expression, conditions that are standard for these genes in Arabidopsis plants, these proteins would facilitate the transit of target proteins to the organelle, functioning as chaperon-like partners. However, under stress conditions favoring their strong overexpression^21^, VQ1 and VQ10 would change their functions from facilitating to scavenging plastid target proteins, preventing efficient import to chloroplast, and thus interfering with the normal organelle function. Nevertheless, more experimental work will be needed to support a speculative functional model like this. When we measured the efficient photosynthetic activity of plants with altered *VQ1* or *VQ10* expression, we found a correlation between enhanced or diminished photosynthesis efficiency and overexpression or amiRNA-downregulated transgenic plants, respectively (Fig. 7A, B). On the other hand, our measurements of different photosynthetic parameters in non-stressed plants as well as in plants undergoing oxidative stress (Fig. 8) known to upregulate *VQ1* and *VQ10* expression, suggest that these proteins likely contribute to improve the efficiency of photosynthesis under stress conditions by reducing energy losses through heat dissipation and by keeping the photosystem reaction centres open. We also investigated whether chloroplast biogenesis and greening were affected in plants with altered *VQ1* or *VQ10* expression. The analysis of greening after de-etiolation revealed reduced chlorophyll levels in both *VQ10ox* overexpressing and in amiRNA lines targeting both *VQ1* and *VQ10* genes (Fig. 7C). Our findings suggest that chloroplast biogenesis and functions, including photosynthesis, could be regulated by VQ1 and VQ10 through mechanisms that are still not fully understood, likely mediated by protein interaction with various key plastid proteins before their import to the organelle.

## Methods

### Plant growth and plasmid constructs

Arabidopsis (*Arabidopsis thaliana*) seeds were surface-sterilized with chlorine gas before sowing on MS (Duchefa Biochemie, The Netherlands) medium plates containing 1% (w/v) sucrose. Seeds from wild type Col-0 and *vq10-H* (SAIL_23_A10C1) genotypes were obtained from the Nottingham Arabidopsis Stock Center (NASC). Plants overexpressing N-terminal 3×HA-tagged or C-terminal -RFP tagged versions of VQ1 or VQ10 were generated by Gateway subcloning of the full-length cDNAs, cloned in *pCR8 TOPO* vector, into either the pAlligator 2 vector^39^ or the *pGWB454* vector^40^. Lines expressing artificial microRNAs (amiRNAs) were generated with the best candidate amiRNA sequences for targeting *VQ1*, *VQ10* or both genes together using the amiRNA Designer tool of the plant small RNA maker suite^41^ (P-SAMS; http://p-sams.carringtonlab.org). The resulting amiRNA inserts were cloned in the *pMDC123SB-AtMIR390a-B/c* binary vector^42,43^. Oligonucleotides used in this work are summarized in Supplementary Table S1. Constructs were used to transform *Agrobacterium tumefaciens* C58 strain. Transformed Agrobacterium was used to genetically transform Col-0 plants by dipping floral organs in a suspension of transformed Agrobacterium^44^ and to select homozygous plants with the suitable selection antibiotic markers. Surface-sterilized seeds were sown in MS medium supplemented with the proper antibiotic or glufosinate herbicide to select transformed resistant seeds or by selecting fluorescent seeds in the case of *pAlligator 2*-based constructs. Plants were grown under photoperiodic conditions (16 h light:8 h darkness; 100 μmol m^−2^ s^−1^) at 21 ℃.

For transient transformation *Nicotiana benthamiana* plants were co-agroinfiltrated with the binary vector-based plasmids in the presence of the silencing suppressor protein P19^45^.

### De-etiolation, chlorophyll quantification, and photosynthetic efficiency

After stratification, the sown seeds on MS medium were exposed to 6 h of light to induce germination and then kept in darkness for 3 days. Etiolated seedlings were subsequently returned to light for 24 h. Samples of whole seedlings (20–100 mg) were collected and extracted with 80% acetone (v/v) for 5 h at 4 ºC in darkness. Absorbance at 647 and 663 nm was measured using a Multiskan GO plate spectrophotometer (Thermo Scientific) with SkanIt Software v.7.1 (Thermo Scientific). Chlorophyll a and b were calculated as previously reported^46^.

Photosynthetic efficiency was calculated from parameters derived from chlorophyll fluorescence measurements in 12–15 day-old in vitro grown seedlings using a Handy FluorCam FC 1000-H with FluorCam7 software. After 20 min darkness incubation, seedlings were exposed to actinic light pulses (21 PAR intensity). Effective quantum yield of Photosystem II (ΦPSII) and maximum quantum yield (Fv/Fm), non-photochemical quenching (NPQ and qN), and proportion of open PSII centres (qP) were determined.

### Visualization of fluorescent-tagged proteins by confocal microscopy

Leaves of *Nicotiana benthamiana* plants expressing VQ1 and VQ10 proteins tagged with RFP at the C-terminus and DXS tagged with GFP and the N-terminus were visualized using an AxioObserver Zeiss LSM 780 confocal microscope. GFP fluorescence was excited at 488 nm with emission detected at 490–527 nm, while RFP fluorescence was excited at 561 nm with emission detected at 588–634 nm emission. Additionally, autofluorescence of chloroplasts, that emits at 674–715 nm after excitation with any of the mentioned wavelengths.

### Yeast two-hybrid binary interactions and library screening

Yeast strains Y187 and Y2HGold, transformed with prey proteins fused to the GAL4 activation domain (AD) and with bait proteins fused to the GAL4 binding domain (BD), respectively, were used to assess protein–protein interactions through mating following the Matchmaker Gold Yeast Two-Hybrid System (Takara Bio) protocol. The coding sequences of *VQ1*, *VQ10*, *VQ24*, *VQ27*, and *VQ32* genes as well as those from *WRKY18*, *WRKY33*, *WRKY40*, and *WRKY75* genes were cloned in *pGADT7-GW* and *pGBKT7-GW* vectors and used to transform Y187 (MATα) and Y2HGold (MATa) yeast strains. Autoactivation controls for the prey clones were conducted using yeast transformed with the empty GAL4-AD plasmid. Diploid yeasts were plated on SD/QDO (–Ade/–His/–Leu/–Trp) to check for protein interactions and on SD/DDO (–Leu–Trp) to verify the successful transformation with both plasmids.

To search for proteins interacting with VQ1, a universal normalized Arabidopsis Mate & PlateTM library (Takara Bio) in Y187 strain was screened by mating with Y2HGold strain transformed with the *pGBKT7-GW-VQ1* plasmid following the above-mentioned protocol with slight modifications. After mating, cultures were plated on 55 large Petri dishes containing SD/QDO (–Ade/–His/–Leu/–Trp) medium and incubated at 28 ºC for 5 days. Colonies were confirmed by plating on SD/QDO (–Ade/–His/–Leu/-Trp) medium supplemented with 40 µg/ml X-α-Gal for blue colony selection. After colony-PCR with specific pGADT7 primers (Supplementary Table S5), the amplified prey sequences were cleaned with ExoSAP-IT Express PCR Product Cleanup (Applied Biosystems) and subjected to Sanger sequencing.

### Protein extraction and immunoprecipitation

Protein extracts from *Nicotiana benthamiana* leaves were prepared from powder obtained in liquid nitrogen, followed by extraction with 50 mM Tris–HCl pH 7, 5 buffer supplemented with 150 mM NaCl, 10% (v/v) de glycerol, 0.15% (v/v) Nonidet P-40, 1% (v/v) protease inhibitor cocktail (Sigma–Merck), 1 mM phenylmethylsulphonyl fluoride (PMSF), and the indicated dithiothreitol (DTT) concentration in each case. After centrifugation at 14,000×*g* for 15 min, the supernatant was used as the total protein extract.

*In planta* protein interactions between DXS-GFP and HA-tagged or RFP-fused versions of VQ1 or VQ10 were analyzed by co-immunoprecipitation using anti-GFP M-270 GFP-Trap^®^ magnetic beads or RFP-Trap Magnetic Agarose (ChromoTek), respectively, and a DynaMag^™^-2 magnetic rack (Life Technologies). The GFP- and HA-tagged co-immunoprecipitated proteins were then analyzed by Western blotting with monoclonal anti-GFP Living ColorsTM (Takara Bio, 1:4000) followed by secondary anti-IGg mouse antibody coupled to HRP (GE HealthCare, 1:10,000), and monoclonal anti-HA-HRP (Thermo Scientific, 1:1000), respectively. The blots were visualized using Amersham ECL Prime (Cytiva) in a chemiluminescence ImageQuantTM 800 system (Cytiva).

### Quantification of isoprenoids

Quantitative analyses of carotenoids, chlorophylls and tocopherols was performed at the Metabolomics Platform of the IBMCP (CSIC-UPV). Isoprenoid metabolites were extracted from 4 mg of lyophilized powder from whole 14 days-old seedlings with methanol. Cantaxanthin was spiked to the samples as an internal control of extraction. Extracts were evaporated using an SpeedVac Concentrator Plus (Eppendorf, Germany) and further dissolved in acetone and filter through 0.2 µm filters. Samples (10 µl) were analyzed by an Agilent series 1200 HPLC system through a C30 reverse phase column (YMC carotenoid, 250 × 4.6 mm × 3 µm from YMC Co., Japan) with three phases of methanol (solvent A); water/methanol (20/80 v/v) supplemented with 0.2% ammonium acetate (solvent B); and tert-methyl buthyl eter (solvent C). Metabolites were separated with the following gradient: 95% of A; isocratic 5% of B for 12 min; 80% A, 5% B, 15% C for 12 min; and then a linear gradient of 30% A, 5% B, 65% C for 30 min at a constant flow rate of 1 ml/min. Detection of carotenoids and chlorophylls was achieved with a photometric diode array (PDA, Santa Clara, USA) at 650 nm and 472 nm, respectively. Tocopherols were detected by fluorescence at 330 nm. Quantification of peaks was performed using Agilent ChemStation software.

### In silico* analyses and predictions*

Cloning and manipulation of plasmids were performed using the Benchling platform (https://www.benchling.com/), and translation to amino acid sequences was conducted with Expasy (https://web.expasy.org/translate/). Protein sequence alignments were carried out with Clustal omega (https://www.ebi.ac.uk/Tools/msa/clustalo/;^47^). Gene Ontology Consortium tools (http://www.geneontology.org;^48^) and PANTHER (http://go.pantherdb.org/;^49^) were utilized to analyze the enrichment of functional categories. Prediction of protein–protein interactions was performed using the iFrag algorithm (http://sbi.imim.es/web/index.php/research/servers/iFrag?;^50^).

## Supplementary Information

Supplementary Information.

## Data Availability

All biological materials are available from the corresponding author upon request. Supporting data not included in the main text of the article are available in the additional information as supplementary files.
