# Supplementary material for: Arabidopsis VQ motif-containing proteins VQ1 and VQ10 interact with plastidial 1-deoxy-D-xylulose-5-phosphate synthase
Source: Sci Rep. 2024 Aug 15;14:18930. doi: 10.1038/s41598-024-70061-x (PMC11666741; doi:10.1038/s41598-024-70061-x)
Supplement: Supplementary file 1 — Supplementary Information. [file 41598_2024_70061_MOESM1_ESM.pdf]

Supplementary data Gayubas et al. Scientific Reports

Title: Arabidopsis VQ motif-containing protein homologues VQ1 and VQ10 interact with plastidial 1-deoxy-D-xylulose-5-phosphate synthase

Authors: Beatriz Gayubas[§], Mari-Cruz Castillo[§], José León[*]

Affiliation: Instituto de Biología Molecular y Celular de Plantas (Consejo Superior de Investigaciones Científicas – Universidad Politécnica de Valencia), 46022 Valencia (Spain)

Figure S1. Subcellular localization of VQ10-RFP and mutated VQ10(C58S)-RFP.

Figure S2. Measurements of NPQ, qN and qP photosynthetic parameters in plants with enhanced or reduced *VQ1* and *VQ10* gene expression.

Figure S3. Original blots/gels of the different panels presented in Figure 3.

Figure S4. Original blots/gels of the different panels presented in Figure 5.

Table S1. Features of VQ proteins and WRKY transcription factors encoded by hypoxia-, NO- and oxidative stress-induced genes.

Table S2. VQ1-interacting proteins identified in the mating-based yeast two hybrid screening of a universal normalized Mate&Plate library.

Table S3. Gene ontology analysis of functional categories over-represented among identified VQ1-interacting proteins.

Table S4. VQ1 interacting proteins with chloroplast localization identified in the Y2H screening.

Table S5. Oligonucleotides used in this work.

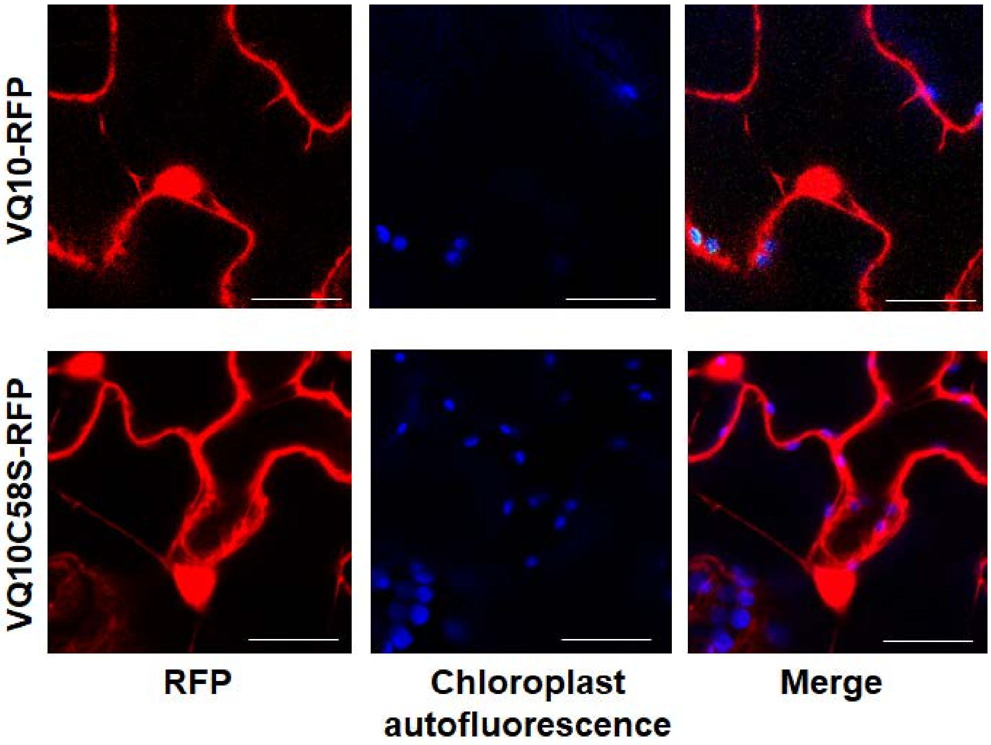

Figure S1. Subcellular localization of VQ10-RFP and mutated VQ10(C58S)-RFP. *Nicotiana benthamiana* leaves were infiltrated with Agrobacterium transformed with *35S:VQ10-RFP* (VQ10-RFP) or *35S:VQ10(C58S)-RFP* (VQ10C58S-RFP) as indicated. Confocal microscopy images of each channel (red for RFP and blue for chloroplast autofluorescence) as well as merge image are shown. Bar size corresponded to 10 mm.

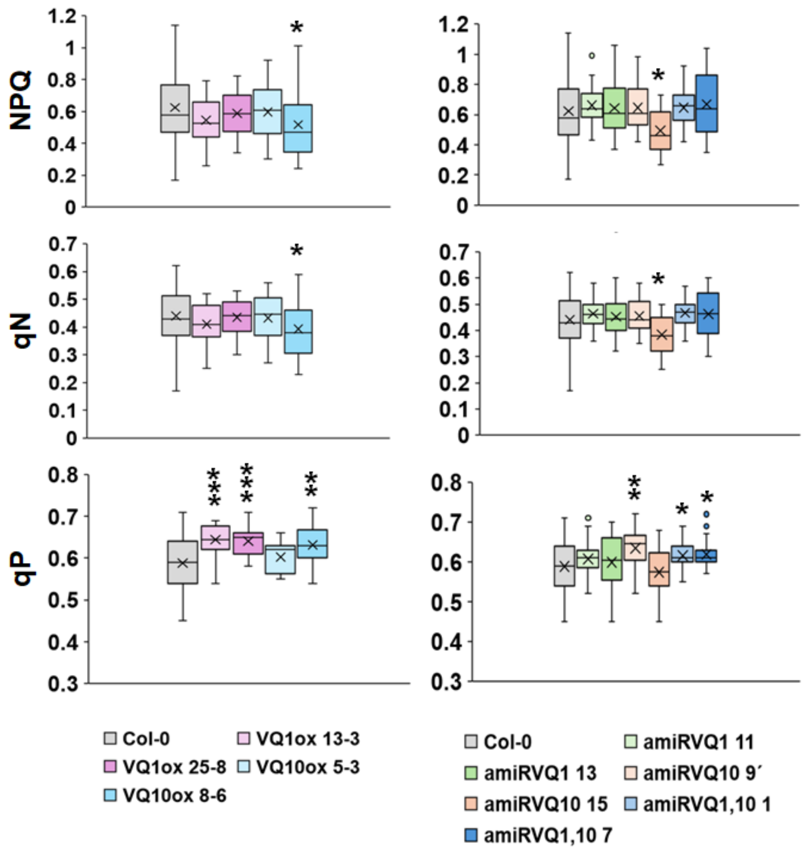

Figure S2. Measurements of NPQ, qN and qP photosynthetic parameters in plants with enhanced or reduced VQ1 and VQ10 gene expression. Parameters were measured after incubation of between 10 and 15 individuals of overexpressing plants (panels in left column) or plants expressing amiRs (panels in the right column) under darkness for 20 min before being exposed to a flash of actinic light (2000 µmol m-2 s-1). Box plots are shown displaying the median (horizontal bar inside the box), and the error interval. Statistical significance was assessed by unpaired t-test comparing each genotype to Col-0. $p<0.05$ *; $p<0.005$ **; and $p<0.001$ ***.

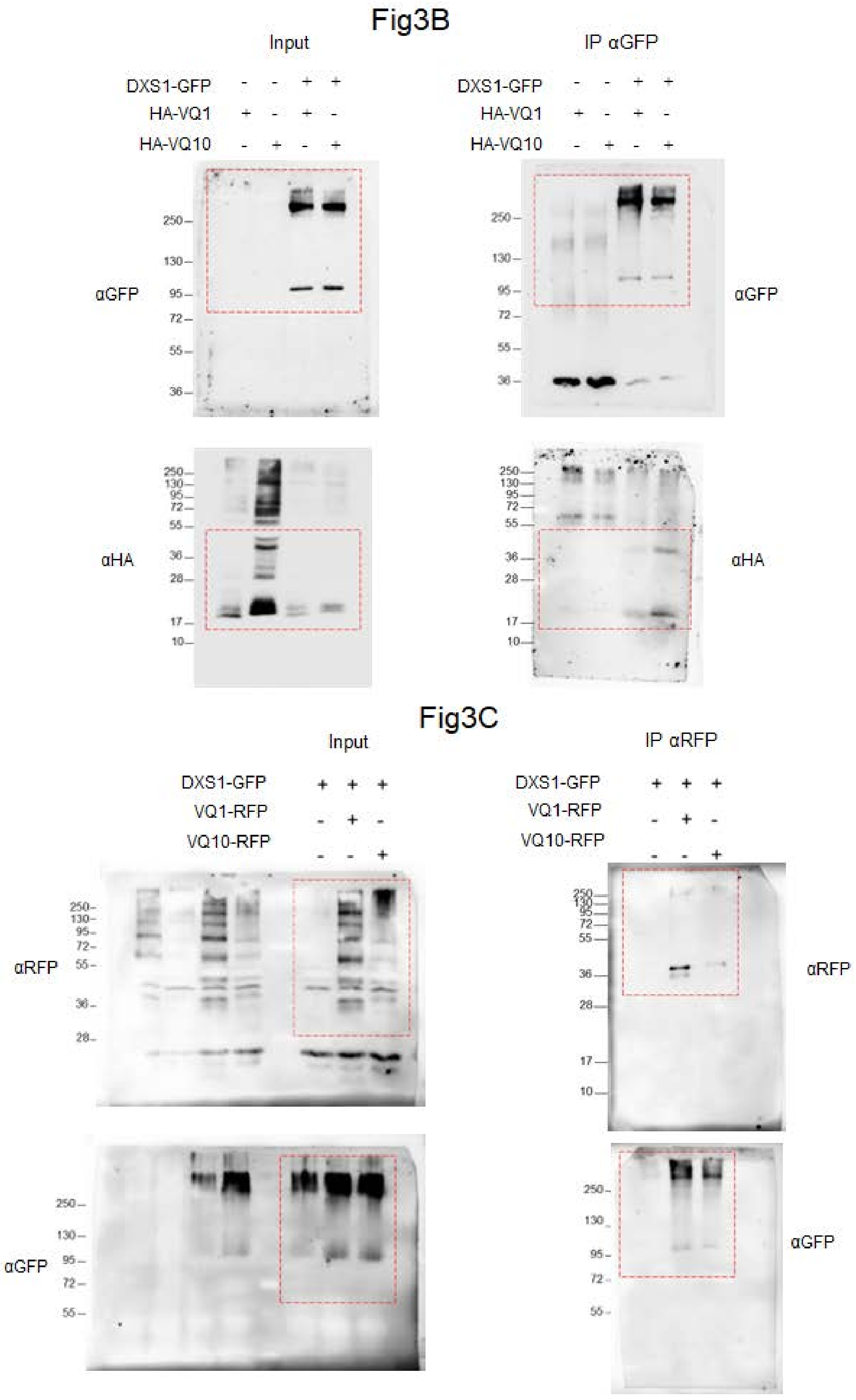

Supplementary Figure 3. Original blots/gels corresponding to Figure 3. Dotted red frames correspond to blots shown in panels B and C of Figure 5. 7.5% and 12.5% PAGE for aGFP and aRFP or aHA, respectively.

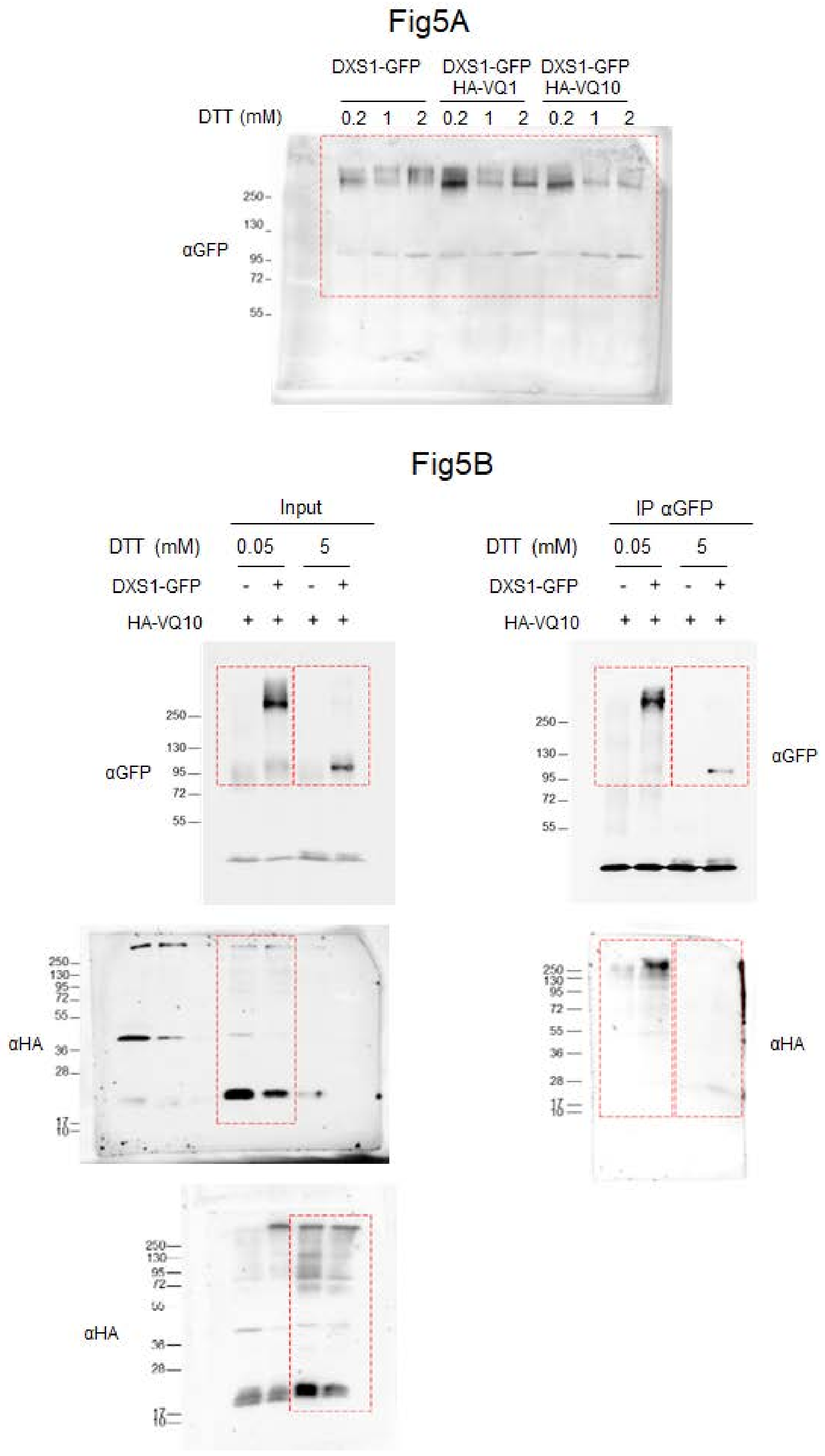

Supplementary Figure 4. Original blots/gels corresponding to Figure 5. Dotted red frames correspond to blots shown in panels A and B of Figure 5. 7.5% and 12.5% PAGE for aGFP and aRFP or aHA, respectively.

Fig5C

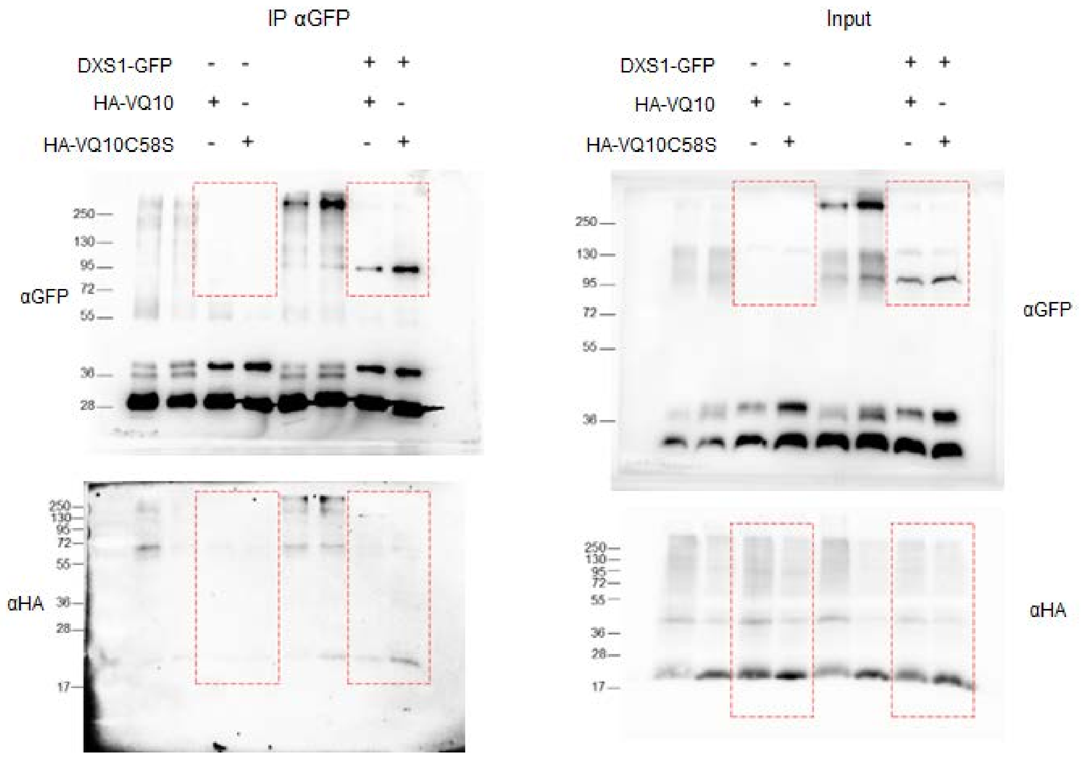

Supplementary Figure 4. Original blots/gels corresponding to Figure 5. Dotted red frames correspond to blots shown in panels A and B of Figure 5. 7.5% and 12.5% PAGE for aGFP and aRFP or aHA, respectively.

Supplementary Table S1. Features of VQ proteins and WRKY transcription factors encoded by hypoxia-, NO- and oxidative stress-induced genes.

AGI loci	Protein	Protein-DNA[(a)]	Protein-RNA[(b)]	Protein-Protein[(c)]	Transcription Regulation[(d)]	Targeting Peptide[(e)]	Subcellular Localization[(f)]
At1g17147	VQ1	NO	YES	YES	NO	Mit	Cyt/Nuc/Chl
At1g78410	VQ10	NO	YES	YES	NO	NO	Cyt/Nuc/Chl
At3g56880	VQ24	NO	NO	YES	YES	NO	Nuc
At4g15120	VQ27	NO	NO	YES	NO	Chl	Mem/Chl/Nuc
At5g46780	VQ32	NO	NO	YES	YES	NO	Nuc
At4g31800	WRKY18	YES	NO	YES	YES	NO	Nuc
At2g38470	WRKY33	YES	NO	YES	YES	Mit	Nuc
At1g80840	WRKY40	YES	NO	YES	YES	NO	Nuc
At5g13080	WRKY75	YES	NO	YES	YES	NO	Nuc

[(a)]DNA binding prediction was performed by DNABIND (https://dnabind.szialab.org/). [(b)]Amino acid residues with probability of being involved in protein-RNA binding were analyzed with DRNApred (http://biomine.cs.vcu.edu/servers/DRNApred/) and PPRInt (http://crdd.osdd.net/raghava/pprint/). [(c)]Positive interaction between proteins was proposed according to Arabidopsis Interaction Viewer database (http://bar.utoronto.ca/interactions/cgi-bin/arabidopsis_interactions_viewer.cgi). [(d)]According to Jing and Lin (2015); Chen and Chen (2002); Birkenbihl et al (2012); Xu et al (2006); Rishmawi et al (2014). [(e)]Prediction of Targeting Peptides was performed by iPSORT (https://ipsort.hgc.jp/). [(f)]Prediction of subcellular localization was performed by Plant-mSubP (http://bioinfo.usu.edu/Plant-mSubP/).

Supplementary Table S2. VQ1-interacting proteins identified in the mating-based yeast two hybrid screening of a universal normalized Mate&Plate library.

AGI	Annotation	Uniprot	Subcellular localization
AT4G18260	Cytochrome b561/ferric reductase transmembrane protein family	B561M	Vacuole
AT3G29330	Zinc finger RNA-binding-like protein	Q6E238; Q6E239	Nucleus
AT3G59280	AtPAM16_MUSE5_PAM16_TXR1__Protein Transporter, Pam16	TM16B	Mitochondria
AT1G70760	CRR23_NdhL__inorganic carbon transport protein-related	NDHL	Chloroplast
AT5G54850	Unknown	Q9FFU5	
AT1G34130	STT3B_staurosporin and temperature sensitive 3-like b	STT3B	ER/Nucleolus
AT5G29000	PHL1__Homeodomain-like superfamily protein	PHL1	Nucleus
AT3G04790	EMB3119__Ribose 5-phosphate isomerase, type A protein	RPI3	Chloroplast
AT5G12960	Proline-tRNA ligase	Q84W43	
AT5G66055	AKRP_EMB16_EMB2036__ankyrin repeat protein	AKRP	Chloroplast
AT2G34070	TBL37__TRICHOME BIREFRINGENCE-LIKE 37	TBL37	Golgi
AT2G35790	Uncharacterized protein	Q9ZQQ1	Mitochondria
AT1G32960	ATSBT3.3_SBT3.3__Subtilase family protein	SBT33	Secreted
AT1G73350	Ankyrin repeat protein	A8MS11;Q84WW0	Nucleus
AT3G62420	ATBZIP53_ basic region/leucine zipper motif 53	BZP53	Nucleus
AT3G08850	ATRAPTOR1B_RAPTOR1_RAPTOR1B__HEAT repeat ;WD domain, G-beta repeat protein protein	RTOR1	Cytoplasm
AT2G32640	LCYB__Lycopene beta/epsilon cyclase protein	F4ITT3;Q0WVA1	Chloroplast
AT4G32140	EamA-like transporter family	O49378	Endomembrane

AT3G20060	UBC19__ubiquitin-conjugating enzyme19	UBC19	Cytop/Nucleus
AT3G51880	AtHMGB1_HMGB1_NFD1__high mobility group B1	F4J5M5;HMGB 1	Nucleus
AT1G75580	SAUR51__SAUR-like auxin-responsive protein family	Q9LR00	Plasma membrane
AT4G18390	TCP2__TEOSINTE BRANCHED 1, cycloidea and PCF transcription factor 2	TCP2	Nucleus
AT4G00100	ATRPS13A_PFL2_RPS13_RPS13A__ribosomal protein S13A	RS132	Cyto, ER, Nucleolus
AT5G24300	ATSS1_SS1__Glycogen/starch synthases, ADP-glucose type	SSY1	Chlorop/Amylop
AT4G18970	GDSL-like Lipase/Acylhydrolase superfamily protein	F4JSC9;GDL65	Secreted
AT1G17720	ATB BETA__Protein phosphatase 2A, regulatory subunit PR55	2ABB	Cytop/Nucleus
AT5G42470	BRISC and BRCA1-A complex member 2	Q5XF81	Cytop/Nucleus
AT5G35630	ATGSL1_GLN2_GS2__glutamine synthetase 2	GLNA2	Chlorop/Mito
AT1G73970	Obscurin-like protein	F4HTS4	
AT5G15090	AtVDAC-3_ATVDAC3_VDAC3__voltage dependent anion channel 3	VDAC3	Plasma memb/Mito
AT3G13130	Unknown transmembrane protein	Q9LK60	Endomembrane
AT3G26510	Octicosapeptide/Phox/Bem1p family protein	Q27GK5;Q6ID88	
AT3G16370	GDSL-like Lipase/Acylhydrolase superfamily protein	APG2	Secreted
AT4G16540	Heat shock protein HSP20/alpha crystallin family	O23498	
ATCG00490	RBCL__ribulose-bisphosphate carboxylases	RBL	Chloroplast
AT2G39990	AteIF3f_EIF2_eIF3F__eukaryotic translation initiation factor 2	EIF3F	Cytoplasm
AT1G11125	Uncharacterized protein	F4I7D8	
AT3G50480	HR4__homolog of RPW8 4	HR4	Endomembrane
AT2G05850	scpl38__serine carboxypeptidase-like 38	SCP38	Secreted
AT4G10110	RNA-binding (RRM/RBD/RNP motifs) family protein	Q8GW25	
AT5G53330	Ubiquitin-associated/translation elongation factor EF1B protein	Q8LG11	Plastid
AT1G79530	GAPCP-1__glyceraldehyde-3-phosphate dehydrogenase of plastid 1	G3PP1	Plstid/Chlorop stroma
AT2G10970	Plant invertase/pectin methylesterase inhibitor superfamily protein	Q9SKH8	
AT4G16140	proline-rich family protein	O23462	Endomembrane
AT5G13100	Uncharacterized protein	Q9FYA0	
AT1G11910	APA1_ATAPA1_AtPaspA1_PaspA1__aspartic proteinase A1	APA1	Vacuole
AT3G23640, AT3G23650	HGL1__heteroglycan glucosidase 1/protein kinase-related	Q93Y12/F4J460	Chloroplast
AT3G06200	Guanylate kinase 3, chloroplastic	GMK3	Chloroplast
AT3G13390	sks11__SKU5 similar 11	Q9LJF2	Chloroplast
AT5G52320	CYP96A4__cytochrome P450, family 96, subfamily A, polypeptide 4	Q9FHC8	Chlorop membrane
AT3G04720	AtPR4_HEL_PR-4_PR4__pathogenesis-related 4	HEVL	Vacuole
AT4G15520	tRNA/rRNA methyltransferase (SpoU) family protein	Q8GYT1	
AT3G06850	BCE2_DIN3_LTA1__2-oxoacid dehydrogenases acyltransferase family protein	ODB2	Mitochondria
AT2G34620	mTERF10__Mitochondrial transcription termination factor family protein	O64685	Mitochondria
AT5G23090	NF-YB13__nuclear factor Y, subunit B13	F4KBG0;F4KB G1;NC2B	Nucleus
AT1G64510	PRPS6__Translation elongation factor EF1B/ribosomal protein S6 family protein	RR6	Plastid/Chlorop
AT5G27030	TPR3__TOPLESS-related 3	F4K2T3;TPR3	Nucleus
AT3G05840	ATSK12_ASK3__Protein kinase superfamily protein	KSG3	Cytop/Cell cortex
AT5G15800/A T3G02310	AGL2_SEP1/AGL4_SEP2__K-box region and MADS-box transcription factor family protein	F4KB90;SEP1/S EP2	Nucleus
AT3G22680	DMS7_RDM1__RNA-DIRECTED DNA METHYLATION 1	RDM1	Nucleus

AT3G47420	AtG3Pp1_ATPS3_G3Pp1_PS3__phosphate starvation-induced gene 3	GLPT1	Endomembrane
AT4G27130	Translation initiation factor SUI1 family protein	SUI11	Cytoplasm
AT4G24460	CLT2__CRT (chloroquine-resistance transporter)-like transporter 2	CLT2	Plastid memb
AT1G06040	BBX24_STO__B-box zinc finger family protein	BBX24; F4IBY7	Nucleus
AT4G26520	AtFBA7_FBA7__Aldolase superfamily protein	ALFC7	Cytoplasm
AT2G19640	ASHR2_SDG39__ASH1-related protein 2_Histone-lysine N-methyltransferase	ASHR2	Nucleus
AT2G20630	PIA1__PP2C induced by AVRRPM1	P2C20	Mitochondria
AT5G20550	2-oxoglutarate (2OG) and Fe(II)-dependent oxygenase superfamily protein	Q8LF12	
AT2G07725	Ribosomal L5P family protein	Q6NMS1	Mitochondria
AT4G26850	GGP_VTC2__mannose-1-phosphate guanylyltransferase (GDP)s	GGAP1	Cytop/Nucleus
AT1G61870	PPR336_rPPR1__pentatricopeptide repeat 336	PPR87	Mito inner memb
AT1G78240	OSU1_QUA2_TSD2__S-adenosyl-L-methionine-dependent methyltransferases superfamily protein	PMTT	Golgi
AT5G50665	Uncharacterized protein	F4KAD6;Q6DB B7	
AT1G70680	Caleosin-related family protein_Probable peroxygenase 5	PXG5	Endomembrane
AT3G59870	Uncharacterized protein	Q9M1Y7	Chlorop stroma
AT3G17510	CIPK1_SnRK3.16__CBL-interacting protein kinase 1	CIPK1	Plasma memb
AT4G00895	ATPase, F1 complex, OSCP/delta subunit protein	Q8W481	
AT1G74260	PUR4__purine biosynthesis 4	PUR4	Chlorop/Mito
AT3G26934	Uncharacterized protein	Q9LJF6	
AT1G30510	ATRFNR2_RFNR2__root FNR 2	FNRR2	Chloroplast
AT5G14260	Rubisco methyltransferase family protein	Q8VZB5	Chlorop stroma
AT2G28315	UXT1__UDP-xylose transporter 1	UXT1	Golgi/ER
AT1G25510	Eukaryotic aspartyl protease family protein	Q9C6M0	
AT4G08950	EXO__EXORDIUM_Phosphate-responsive 1 family protein	EXO	Secreted
AT1G76920	SKP1-interacting partner 15_F-box family protein	SKI15	Nucleus
AT3G01230	Splicing regulatory glutamine/lysine-rich-like protein	Q9MAD2	
AT2G04790	PTB domain engulfment adapter	F4IFB5;F4IFB6; Q6ICW4	
AT5G09550	GDI__GDP dissociation inhibitor family protein / Rab GTPase activator family protein	GDI	Cytop/Secreted
AT4G04078	Uncharacterized protein of 35 aa	B3H7E1	
AT1G47380	Protein phosphatase 2C family protein	P2C12	
AT1G52540	Protein kinase superfamily protein	Q8LDB7	Plasma memb/Plasmodesm
AT3G18060	Actin-interacting protein 1-2_transducin family protein / WD-40 repeat family protein	AIP12	Cytop/Cytoskel
AT2G44160	MTHFR2__methylenetetrahydrofolate reductase 2	MTHR2	Cytoplasm
AT1G12050	AtFAH_FAH_SSCD1__fumarylacetoacetase, putative	FAH	Cytop/Secreted
AT2G25830	YebC-related probable transcriptional regulatory protein	U082	Chlorop/Vacuole
AT4G35060	HIPP25__Heavy metal transport/detoxification superfamily protein	HIP25	Plasma memb/Plasmod
AT3G13400	sks13__SKU5 similar 13	Q9LJF1	Secreted
AT3G02740	Eukaryotic aspartyl protease family protein	Q9M8R6	Cytop/Plasma memb/Plasmodesm
AT3G54830	Amino acid transporter AVT1B_Transmembrane amino acid transporter family protein	AVT1B	Membrane
AT5G37475	Translation initiation factor eIF3 subunit	Q8GRX2	Cytoplasm
AT3G54050	Fructose-1,6-bisphosphatase 1, chloroplastic_cfbp1_HCEF1__high cyclic electron flow 1	F16P1	Chlorop stroma
AT5G43060	RD21B__Granulin repeat cysteine protease family protein	RD21B	ER/Vacuole/Secreted

AT5G19620	ATOEP80_EMB213_OEP80_TOC75-V__outer envelope protein of 80 kDa	OEP80	Chlorop outer memb
AT2G44410	RING/U-box superfamily protein	Q6NLR3	ER/Nucleus
AT1G03250	R3H domain protein	F4I0U6;Q8L3X7	
AT3G27220	HUP6_Hypoxia response unknown protein 6_Galactose oxidase/kelch repeat superfamily protein	Y3272	Endomembrane
AT4G13220	Uncharacterized protein	Q6NLB6	Endomembrane
AT5G04140	FD-GOGAT_ glutamate synthase 1	GLTB1	Chlorop stroma/Mito
AT3G51130	PHAF1 protein	Q9SD33	
AT5G04590	SIR__sulfite reductase	Q9LZ66;SIR	Chlorop stroma
AT2G30490	ATC4H_C4H_CYP73A5_REF3__cinnamate-4-hydroxylase	TCMO	Endomembrane
AT1G23820	SPDS1__spermidine synthase 1	F4I7M5;SPDS1	
AT3G54130	Ataxin-3 homolog_Josephin family protein_Transcription repressor	ATX3H	Nucleus
AT5G23730	EFO2_RUP2__Transducin/WD40 repeat-like superfamily protein	RUP2	Cytop/Nucleus
AT4G13540	Golgin family A protein	Q9T0H4	Peroxisome
AT4G23840	Leucine-rich repeat (LRR) family protein	Q8L4C7	
AT3G23200	CASPL5B3__Uncharacterised protein family (UPF0497)	CSPLG	Plasma membrane
AT3G54210	PRPL17__Ribosomal protein L17 family protein	RK17	Chloroplast
AT2G46280	TIF3I1_TRIP-1_TRIP1__TGF-beta receptor interacting protein 1	EIF3I;F4II65	Cytoplasm
AT3G13110	ATSERAT2;2_SAT-1_SAT-A_SAT-M_SAT3_SERAT2;2__serine acetyltransferase 2;2	SAT3	Mitochondria
AT3G49360	PGL2__6-phosphogluconolactonase 2	6PGL2	Cytoplasm
AT5G46180	DELTA-OAT__ornithine-delta-aminotransferase mitochondrial	OAT	Mitochondria
AT1G17350	Probable complex I intermediate-associated protein 30_NADH:ubiquinone oxidoreductase intermediate-associated protein 30	CIA30;F4I7I6	Mitochondria
AT5G24420	PGL5__6-phosphogluconolactonase 5	6PGL5	Cytoplasm
AT5G20900	JAZ12_TIFY3B__jasmonate-zim-domain protein 12	TIF3B	Nucleus
AT1G32580	Multiple organellar RNA editing factor 5, chloroplastic/mitochondrial MORF5	MORF5	Chlorop/Mito
AT1G73750	Uncharacterised conserved protein UCP031088, alpha/beta hydrolase	Q8W568	
AT5G15140	Galactose mutarotase-like superfamily protein_Aldose 1-epimerase family protein	Q9LXG7	
AT5G58110	chaperone binding;ATPase activators	Q9FGT3	Cytoplasm
AT1G20696	HMGB3_NFD03_NFD3__high mobility group B3	HMGB3	Cytop/Nucleus
AT4G36500	Uncharacterized protein	O23231	
AT3G17330	ECT6__evolutionarily conserved C-terminal region 6	A8MPR6;Q1JPL5	Cytoplasm
AT5G16390	BCCP_BCCP-1_BCCP1 chloroplastic acetylcoenzyme A carboxylase 1	BCCP1;F4KE21	Chloroplast
AT5G65840	Thioredoxin superfamily protein	Q8L5Y8	Chlorop stroma
AT2G36250	ATFTSZ2-1_FTSZ2-1__Tubulin/FtsZ family protein	FTZ21	Chlorop stroma&thylak
AT1G04140	Transducin family protein / WD-40 repeat family protein	F4I458;Q94C94	
AT3G51250	Senescence/dehydration-associated protein-related	SDEH3	Plasma memb/Plasmod
AT3G32930	6,7-dimethyl-8-ribityllumazine synthase	Q9LHC4	Chlorop envelope
AT2G33590	AtCRL1_CRL1__NAD(P)-binding Rossmann-fold superfamily protein	O22809	Chloroplast
AT1G15030	Uncharacterized protein	Q9M9Q2	
AT3G26900	ATSKL1_SKL1__shikimate kinase like 1	SKL1	Chloroplast
AT1G13440	GAPC-2_GAPC2__glyceraldehyde-3-phosphate dehydrogenase C2	F4HQT1;G3PC2	Cytop/Nucleus
AT5G50100	DCC1__Putative thiol-disulphide oxidoreductase DCC	Y5010	Chloroplast
AT3G07525	ATATG10_ATG10__autophagocytosis-associated family protein	ATG10	Endomembrane

AT5G47810	PFK2__phosphofructokinase 2	PFKA2	Cytoplasm
AT5G49030	OVA2__tRNA synthetase class I (I, L, M and V) family protein	F4K4Q2;SYIM	
AT1G56700	Peptidase C15, pyroglutamyl peptidase I-like	A8MSE7;Q9FXC0	Cytoplasm
AT5G18650	MIEL1__CHY-type/CTCHY-type/RING-type Zinc finger protein	MIEL1	Cytop/Nucleus
AT2G31610	40S ribosomal protein S3-1	RS31	Cytop/nuc/Chlorop/perox/plasmod/vacuole
AT5G08100	ASPGA1__N-terminal nucleophile aminohydrolases (Ntn hydrolases) superfamily protein	ASPGA;F4K9K7	
AT3G27930	AtOM47_OM47	F4IXS2;Q8W106	Mit outer memb
AT1G55880	Pyridoxal-5'-phosphate-dependent enzyme family protein	F4I3G8;Q6NKY5	Endomembrane
AT5G15025	Unknown	Q9LFQ2	Nucleus
AT2G42910	AtPRS4_PRS4__Phosphoribosyltransferase family protein	KPRS4	Cytop/Plasma memb/Plasmodesm
AT4G04460	AtPaspA3_PaspA3__Saposin-like aspartyl protease family protein	APA3;F4JGD2	Secreted
AT3G17900	Uncharacterized protein	Q9ASZ4	transGolgi
AT4G14930	Survival protein SurE-like phosphatase/nucleotidase	Q8LAM2	Cytoplasm
AT2G34860	PHOTOSYSTEM I ASSEMBLY 2, chloroplastic_EDA3_PSA2__DnaJ/Hsp40 cysteine-rich domain superfamily protein	PSA2	Chloroplast
AT1G78450	SOUL heme-binding family protein	Q9SYN6	Anatomical entity
AT1G74070	Cyclophilin-like peptidyl-prolyl cis-trans isomerase family protein	CP26B	Chloroplast
AT1G53400	Ubiquitin domain-containing protein	Q9MAG2	
AT1G56500	SUPPRESSOR OF QUENCHING 1, chloroplastic_SOQ1__haloacid dehalogenase-like hydrolase family protein	SOQ1	Chlorop thylakoid
AT4G28556	RIC7__PAK-box/P21-Rho-binding family protein	RIC7	Cyt/Nuc/Plasma memb
AT3G55380	UBC14__ubiquitin-conjugating enzyme 14	F4IWU7;UBC14	
AT5G58680	ARM repeat superfamily protein	Q8LGC7	Cytop/Nucleus
AT1G42960	Inner membrane localized protein	Q9C7S3	Chlorop/Nucleus
AT4G17560	50S Ribosomal protein L19-1	RK191	Chloroplast
AT2G42230	C-CAP/cofactor C-like domain-containing protein	F4IN01;Q66GP5	Cytop/Cytoskel
AT4G02195	ATSYP42_ATTLG2B_SYP42_TLG2B__syntaxin of plants 42	SYP42	Golgi
AT1G19800	ABCI14_TGD1__trigalactosyldiacylglycerol 1	TGD1	Chlorop inner memb
AT5G12040	Omega-amidase, chloroplastic_Nitrilase/cyanide hydratase and apolipoprotein N-acyltransferase family protein	NILP3	Chloroplast
AT1G79500	AtkdsA1_KDO8PS__Aldolase-type TIM barrel family protein	KDSA1	Cytoplasm
AT4G15560	AtCLA1_CLA_CLA1_DEF_DXPS2_DXS_DXS1__Deoxyxylulose-5-phosphate synthase	DXS	Chlorop stroma
AT2G05790	O-Glycosyl hydrolases family 17 protein	F4IHD3	Plasma memb
AT3G46520	ACT12__actin-12	ACT12	Cytop/Cytoskel
AT5G26990	DEHYDRATION-INDUCED 19 homolog 6	DI196	Nucleus
AT3G55770	WLIM2b__GATA type zinc finger transcription factor family protein	F4IY33;WLI2B	Cytop/Cytoskel
AT4G18810	NAD(P)-binding Rossmann-fold superfamily protein	F4JRN8;Q8VYA4	Cytop/Chlorop/vacuole
AT4G28610	AtPHR1_PHR1__phosphate starvation response 1	PHR1	Nucleus
AT2G40330	PYL6_RCAR9__PYR1-like 6	PYL6	Cytop/Nuc/Plasma memb
AT3G18050	GPI-anchored protein	Q9LV36	Endomembrane
AT5G28020	Bifunctional L-3-cyanoalanine synthase/cysteine synthase D2	CYSD2;F4K5T1	Cytoplasm
AT2G29400	PP1-AT_TOPP1__type one protein phosphatase 1	PP11	Cytop/Nucleus
AT5G37600	ATGLN1;1_ATGSR1_GLN1;1_GSR 1__Glutamine synthetase cytosolic isozyme 1-1	GLN11	Cytoplasm
AT1G54030	ERMO3_GOLD36_MVP1_NUC__GDSL-like Lipase/Acylhydrolase superfamily protein	GDL21	ER/Vacuole

AT5G22810	GDSL-like Lipase/Acylhydrolase superfamily protein	GDL78	Secreted
AT4G26930	AtMYB97_MYB97__myb domain protein 97	MYB97	Nucleus
AT4G33520	AtHMAC6_HMA6_PAA1_PCH1__P-type ATP-ase 1	F4JIZ4;HMA6	Endomembrane
AT3G04280	ARR22_RR22__response regulator 22	ARR22	Nucleus
AT1G74880	NDH-O_NdhO__NAD(P)H:plastoquinone dehydrogenase complex subunit O	NDHO	Chlorop thylakoid
AT2G39720	Probable E3 ubiquitin-protein ligase RHC2A_RHC2A__RING-H2 finger C2A	RHC2A	Cytoplasm
AT3G59980	Nucleic acid-binding, OB-fold-like protein	Q9M1X8	Nucleus
AT3G02640	Uncharacterized protein	Q9M878	Endomembrane
AT1G56010	anac021_ANAC022_NAC1__NAC domain containing protein 1	NAC22	Nucleus
AT4G35220	CYCLASE2__Cyclase family protein	CYL2	Secreted/Endomembrane
AT5G27280	Zim17-type zinc finger protein	O04646	Mitochondria
AT4G22930	DHOASE_PYR4__pyrimidin 4	PYRC	Mitochondria
AT3G20510	FATTY ACID EXPORT 6_FAX6__Transmembrane proteins 14C	FAX6	Endomembrane
AT2G34410	REDUCED WALL ACETYLATION 3 RWA3__O-acetyltransferase family protein	RWA3	Golgi
AT2G05632	Uncharacterized protein of 52 aa with 4C	Q0WU16	
AT3G11830	T-complex protein 1 subunit eta TCP-1/cpn60 chaperonin family protein	TCPH	Cytoplasm
AT5G20500	Glutaredoxin-C4	GRXC4	Cytoplasm
AT4G13270	Late embryogenesis abundant (LEA) hydroxyproline-rich glycoprotein family	Q52K84	Endomembrane
AT5G46210	ATCUL4_CUL4__cullin4	CUL4	Nucleus
AT5G59840	AtRabE1b__Ras-related small GTP-binding family protein	Q9FJF1	Plasma memb/Plasmod/Plastid
AT2G44745	AtWRKY12_WRKY12__WRKY family transcription factor	WRK12	Nucleus
AT4G05450	Adrenodoxin-like protein 1, mitochondrial_ATMFDX1_MFDX1__mitochondrial ferredoxin 1	MFDX1	Mitochondria
AT3G46970	ATPHS2_PHS2__alpha-glucan phosphorylase 2	PHS2	Cytoplasm
AT5G18260	RING/U-box superfamily protein	Q9FK45	
AT3G27810	ATMYB21_ATMYB3_MYB21__myb domain protein 21	MYB21	Nucleus
AT1G24480	S-adenosyl-L-methionine-dependent methyltransferases superfamily protein	Q9FYL5	Endomembrane
AT1G75280	Isoflavone reductase homolog P3_NmrA-like negative transcriptional regulator family protein	IFRH	Cytoplasm
AT5G15030	Paired amphipathic helix (PAH2) superfamily protein	Q9LFQ2	Nucleus
AT4G26000	PEP__RNA-binding KH domain-containing protein PEPPER	PEP	Nucleus
AT2G20360	NADH dehydrogenase [ubiquinone] 1 alpha subcomplex subunit 9, mitochondrial	NDUA9	Mitochondria
AT3G01480	Peptidyl-prolyl cis-trans isomerase_ATCYP38_CYP38__cyclophilin 38, chloroplastic	CYP38	Chlorop thylakoid
AT4G32770	ATSDX1_VTE1__tocopherol cyclase, chloroplast / vitamin E deficient 1 (VTE1) / sucrose export defective 1 (SXD1)	TOCC	Chloroplast/plastoglob
AT2G36530	ENO2_LOS2__Enolase	ENO2	Cytop/Nuc/Mito om
AT1G58235	Uncharacterized protein of 74 aa	Q93ZI5	
AT2G21385	AtCGLD11_BFA3_CGLD11	A8MQF3;F4IGM4;Q94AU3	Chlorop/Mito
AT4G32520	AtSHMT3_SHM3__serine hydroxymethyltransferase 3	GLYP3	Chloroplast
AT3G54110	ATPUMP1_ATUCP1_PUMP1_UCP_UCP1__plant uncoupling mitochondrial protein 1	PUMP1	Mitochondria im
AT5G24530	DOWNY MILDEW RESISTANCE 6_DMR6__2-oxoglutarate (2OG) and Fe(II)-dependent oxygenase superfamily protein	DMR6	
AT1G25230	Purple acid phosphatase 4	PPA4	Secreted
AT3G25900	ATHMT-1_HMT-1__Homocysteine S-methyltransferase family protein	F4JBA7;F4JBA8;HMT1	

AT1G60010	D-ribose-binding periplasmic protein	Q9ZUJ2	
AT1G34000	Light-harvesting complex-like protein OHP2, chloroplastic__one-helix protein 2	OHP2	Chlorop thylakoid
AT4G35550	ATWOX13_HB-4_WOX13__WUSCHEL related homeobox 13	WOX13	Nucleus
AT5G37478	TPX2 (targeting protein for Xklp2) protein family	F4K773	Cytop/Cytoskel
AT2G26340	Uncharacterized protein	F4IUI9;F4IUJ0	Chlorop thylakoid/Peroxis
AT2G30170	PBCP__Protein phosphatase 2C protein 26	P2C26	Chloroplast
AT4G00570	NAD-ME2__NAD-dependent malic enzyme 2	MAO2	Mitochondria
AT1G80070	PRP8_SUS2__Pre-mRNA-processing-splicing factor 8A	PRP8A	Nucleus
AT1G59900	Pyruvate dehydrogenase E1 component subunit alpha-1, mitochondrial	ODPA1	Mitochondria
AT1G53000	3-deoxy-manno-octulosonate cytidylyltransferase, mitochondrial	KDSB	Mitochondria om
AT3G06483	ATPDHK_PDK__pyruvate dehydrogenase kinase mitochondrial	PDK	Mitochondria
AT1G23560	Domain of unknown function (DUF220)	Q9ZUD8	
AT4G01970	Probable galactinol--sucrose galactosyltransferase 4_RS4_STS__stachyose synthase	RFS4	
AT5G05010	Coatomer subunit delta_Clathrin adaptor complexes medium subunit family protein	COPD	Cytop/Golgi
AT4G39400	ATBRI1_BIN1_BRI1_CBB2_DWF2__Leucine-rich receptor-like protein kinase family protein	BRI1	Plasma memb/Endomemb
AT5G42030	ABIL4__ABL interactor-like protein 4	ABIL4	Cytop/Cytoskel
AT4G03210	XTH9__xyloglucan endotransglucosylase/hydrolase 9	F4JI68;XTH9	Secreted
AT5G47310	PPPDE putative thiol peptidase family protein_Deubiquitinase	Q9LVS8	
AT4G26970	ACO2__aconitase 2 mitochondrial	ACO2M	Mitochondria
AT3G09630	SAC56__Ribosomal protein L4/L1 family	Q2V3X4;RL4A	Cytop/Ribosome
AT1G65840	ATPAO4_PAO4__polyamine oxidase 4	PAO4	Peroxisome
AT1G75460	ATP-dependent protease La (LON) domain protein	Q9FWT4	Chloroplast
AT2G41410	Probable calcium-binding protein CML35	CML35	Cytoplasm
AT2G13570	NF-YB7__nuclear factor Y, subunit B7	NFYB7	Nucleus
AT3G55290	SDRd__NAD(P)-binding Rossmann-fold superfamily protein	F4JFB9;Q94AL3	Peroxisome
AT5G40200	DEG9_DegP9__DegP protease 9	DEGP9	Nucleus
AT4G31990	AAT3_ASP5_ATAAT1__aspartate aminotransferase 5	AAT5;B9DG21;F4JTH0	Chlorop/Amylop
AT2G36000	EMB3114__Mitochondrial transcription termination factor family protein	Q8S8E4;Q9SJ50	Mitochondria
AT1G24050	RNA-processing, Lsm domain	Q8L466	
AT2G23080	Casein kinase II subunit alpha-3	CSK23	Cytop/Nucleus
AT5G63980	SAL1 phosphatase_ALX8_AtFRY1_ATSAL1_FRY1_HOS2_RON1_SAL1_SUPO1__Inositol monophosphatase family protein	DPNP1	
AT5G20350	Protein S-acyltransferase 24_TIP1__Ankyrin repeat family protein with DHHC zinc finger domain	ZDH22	Golgi
AT1G27770	ACA1_PEA1__autoinhibited Ca2+-ATPase 1	ACA1;F4HUS8	Endomembrane
AT3G13510	Protein of Unknown Function (DUF239)	Q9LJE0	
AT5G58950	Protein kinase superfamily protein	Q9FIL6	
AT1G76450	PsbP domain-containing protein 3, chloroplastic_Photosystem II reaction center PsbP family protein	PPD3	Chlorop thylakoid
AT5G45130	Ras-related protein RABF2a_ATRABF2A_RAB-F2A_RAB5A_RABF2A_RHA1__RAB homolog 1	RAF2A	Endomembrane
AT2G33255	Haloacid dehalogenase-like hydrolase (HAD) superfamily protein	GPPL3	Chlorop/Cytop/ER
AT5G07020	MAINTENANCE OF PSII UNDER HIGH LIGHT 1 MPH1__proline-rich family protein	MPH1	Chlorop thylakoid
AT3G12480	NF-YC11__nuclear factor Y, subunit C11	Q9LHG0	Nucleus
AT5G00480	Uncharacterized protein	A0A178ULA0	

AT5G14450	GDSL-like Lipase/Acylhydrolase superfamily protein	GDL76	Secreted
AT1G12910	ATAN11_LWD1__Transducin/WD40 repeat-like superfamily protein	LWD1	Nucleus
AT1G66250	Glucan endo-1,3-beta-glucosidase 2_O-Glycosyl hydrolases family 17 protein	E132	Plasma membrane
AT1G17620	Late embryogenesis abundant (LEA) hydroxyproline-rich glycoprotein family	Q9LNP3	Nucleus/Plasma memb/Plasmod
AT4G16510	YbaK/aminoacyl-tRNA synthetase-associated domain	Q940I4	
AT2G43810	LSM6B__Small nuclear ribonucleoprotein family protein	LSM6B	Cytop/Nucleus
AT1G13270	MAP1B_MAP1C__methionine aminopeptidase 1B chloroplastic	F4HQQ8;MAP1B	Chloroplast
AT1G72160	Patellin-3 PATL3__Sec14p-like phosphatidylinositol transfer family protein	PATL3	Endomembrane/Cytop
AT2G39460	60S ribosomal protein L23a-1	R23A1	Cytop Ribosome/Golgi/Nucleol/Secreted/plasmod
AT4G18640	MALE DISCOVERER 2 MDIS2_MRH1__Leucine-rich repeat protein kinase family protein	MDIS2	Endomembrane
AT5G39740	60S ribosomal protein L5-2	RL52	Cytop/Nuc/Nucleol
AT3G25050	AtXTH3_XTH3__xyloglucan endotransglucosylase/hydrolase 3	XTH3	Secreted
AT5G09620	Octicosapeptide/Phox/Bem1p family protein	Q9LXC6	Plasma membrane
AT5G22420	FAR7__fatty acid reductase 7	FACR7	Endomembrane
AT1G28660	GDSL-like Lipase/Acylhydrolase superfamily protein	GDL12	Secreted
AT5G47030	ATPase, F1 complex, delta/epsilon subunit mitochondrial	ATP4	Mitochondria
AT4G32551	LUG_RON2__Transcriptional corepressor LEUNIG_LisH dimerisation motif;WD40/YVTN repeat-like-containing domain	F4JUD2;LEUNG	Nucleus
AT3G60450	Phosphoglycerate mutase family protein	Q9M214	
AT3G62550	Adenine nucleotide alpha hydrolases-like superfamily protein	Q93W91	Vacuole
AT2G35810	Ureidoglycolate hydrolase	Q8VZP3	
AT4G25670	Stress response NST1-like protein	Q94BX3	

Arabidopsis Genome Initiative (AGI) and UniProt codes of the prey clones identified in the Y2H screening using VQ1 as bait. Subcellular localization was indicated according to UniProt.

Supplementary Table S3. Gene ontology analysis of functional categories over-represented among identified VQ1-interacting proteins.

**GO biological process complete**	**Arabidopsis thaliana - REFLIST (27430)**	**upload_1 (280)**	**upload_1 (expected)**	**upload_1 (fold Enrichment)**	**upload_1 (raw P-value)**	**upload_1 (FDR)**
generation of precursor metabolites and energy (GO:0006091)	407	22	4.15	5.3	5.93E-10	3.49E-06
small molecule metabolic process (GO:0044281)	2857	62	29.16	2.13	1.26E-08	3.70E-05
cellular process (GO:0009987)	14852	191	151.61	1.26	2.24E-06	3.29E-03
pyruvate metabolic process (GO:0006090)	81	8	0.83	9.68	3.42E-06	4.03E-03
alpha-amino acid metabolic process (GO:1901605)	368	16	3.76	4.26	2.17E-06	4.25E-03
cellular metabolic process (GO:0044237)	8829	127	90.12	1.41	6.05E-06	4.45E-03
nucleotide metabolic process (GO:0009117)	313	14	3.2	4.38	6.92E-06	4.53E-03
carboxylic acid metabolic process (GO:0019752)	1537	36	15.69	2.29	5.42E-06	4.56E-03
organonitrogen compound metabolic process (GO:1901564)	5422	88	55.35	1.59	5.22E-06	5.12E-03
nucleoside phosphate metabolic process (GO:0006753)	324	14	3.31	4.23	1.01E-05	5.92E-03

glucose metabolic process (GO:0006006)	46	6	0.47	12.78	1.39E-05	7.44E-03
photosynthesis (GO:0015979)	210	11	2.14	5.13	1.69E-05	8.31E-03
organonitrogen compound biosynthetic process (GO:1901566)	1431	33	14.61	2.26	1.86E-05	8.40E-03
alpha-amino acid biosynthetic process (GO:1901607)	175	10	1.79	5.6	2.02E-05	8.51E-03
nucleobase-containing small molecule metabolic process (GO:0055086)	415	15	4.24	3.54	3.63E-05	1.19E-02
cellular amino acid biosynthetic process (GO:0008652)	190	10	1.94	5.16	3.94E-05	1.22E-02
cellular amino acid metabolic process (GO:0006520)	566	18	5.78	3.12	3.16E-05	1.24E-02
organic acid metabolic process (GO:0006082)	2088	42	21.31	1.97	3.59E-05	1.24E-02
oxoacid metabolic process (GO:0043436)	1953	40	19.94	2.01	3.41E-05	1.25E-02
glutamine family amino acid metabolic process (GO:0009064)	59	6	0.6	9.96	5.06E-05	1.42E-02
glutamine metabolic process (GO:0006541)	17	4	0.17	23.05	5.33E-05	1.43E-02
ribose phosphate metabolic process (GO:0019693)	238	11	2.43	4.53	5.05E-05	1.49E-02
monosaccharide metabolic process (GO:0005996)	127	8	1.3	6.17	7.20E-05	1.57E-02
metabolic process (GO:0008152)	10826	144	110.51	1.3	6.13E-05	1.57E-02
dicarboxylic acid metabolic process (GO:0043648)	92	7	0.94	7.45	6.73E-05	1.58E-02
response to light stimulus (GO:0009416)	2003	40	20.45	1.96	7.00E-05	1.59E-02
response to cadmium ion (GO:0046686)	92	7	0.94	7.45	6.73E-05	1.65E-02
ATP metabolic process (GO:0046034)	97	7	0.99	7.07	9.19E-05	1.80E-02
biosynthetic process (GO:0009058)	3327	57	33.96	1.68	1.01E-04	1.80E-02
response to radiation (GO:0009314)	2045	40	20.87	1.92	8.72E-05	1.83E-02
purine nucleotide metabolic process (GO:0006163)	211	10	2.15	4.64	9.09E-05	1.84E-02
response to abiotic stimulus (GO:0009628)	4114	67	41.99	1.6	9.98E-05	1.84E-02
sulfur amino acid biosynthetic process (GO:0000097)	41	5	0.42	11.95	9.93E-05	1.89E-02
organic substance biosynthetic process (GO:1901576)	3119	54	31.84	1.7	1.38E-04	2.32E-02
primary metabolic process (GO:0044238)	8191	114	83.61	1.36	1.34E-04	2.32E-02
purine ribonucleoside triphosphate metabolic process (GO:0009205)	105	7	1.07	6.53	1.46E-04	2.39E-02
photosynthesis, light reaction (GO:0019684)	142	8	1.45	5.52	1.50E-04	2.39E-02
purine-containing compound metabolic process (GO:0072521)	231	10	2.36	4.24	1.84E-04	2.65E-02
ribonucleotide metabolic process (GO:0009259)	229	10	2.34	4.28	1.72E-04	2.67E-02
purine nucleoside triphosphate metabolic process (GO:0009144)	109	7	1.11	6.29	1.82E-04	2.68E-02
monosaccharide biosynthetic process (GO:0046364)	47	5	0.48	10.42	1.79E-04	2.70E-02
organophosphate metabolic process (GO:0019637)	599	17	6.11	2.78	1.97E-04	2.77E-02
small molecule biosynthetic process (GO:0044283)	898	22	9.17	2.4	2.59E-04	3.47E-02
glutamate metabolic process (GO:0006536)	27	4	0.28	14.51	2.59E-04	3.54E-02
sulfur amino acid metabolic process (GO:0000096)	52	5	0.53	9.42	2.77E-04	3.54E-02

ribonucleoside triphosphate metabolic process (GO:0009199)	117	7	1.19	5.86	2.75E-04	3.59E-02
glutamine family amino acid biosynthetic process (GO:0009084)	29	4	0.3	13.51	3.31E-04	3.75E-02
glycolytic process (GO:0006096)	54	5	0.55	9.07	3.26E-04	3.76E-02
photosynthetic electron transport chain (GO:0009767)	53	5	0.54	9.24	3.01E-04	3.77E-02
ATP generation from ADP (GO:0006757)	54	5	0.55	9.07	3.26E-04	3.84E-02
purine ribonucleotide metabolic process (GO:0009150)	203	9	2.07	4.34	3.26E-04	3.91E-02
sulfur compound biosynthetic process (GO:0044272)	160	8	1.63	4.9	3.25E-04	3.98E-02
purine nucleoside diphosphate metabolic process (GO:0009135)	56	5	0.57	8.75	3.81E-04	4.08E-02
hexose metabolic process (GO:0019318)	88	6	0.9	6.68	3.90E-04	4.10E-02
purine ribonucleoside diphosphate metabolic process (GO:0009179)	56	5	0.57	8.75	3.81E-04	4.15E-02
electron transport chain (GO:0022900)	125	7	1.28	5.49	4.02E-04	4.15E-02
ADP metabolic process (GO:0046031)	56	5	0.57	8.75	3.81E-04	4.23E-02
L-serine metabolic process (GO:0006563)	31	4	0.32	12.64	4.17E-04	4.24E-02
organic substance metabolic process (GO:0071704)	9807	129	100.11	1.29	4.37E-04	4.36E-02
nucleoside triphosphate metabolic process (GO:0009141)	128	7	1.31	5.36	4.60E-04	4.51E-02
NADH dehydrogenase complex assembly (GO:0010257)	33	4	0.34	11.87	5.18E-04	4.92E-02
monocarboxylic acid metabolic process (GO:0032787)	655	17	6.69	2.54	5.34E-04	4.99E-02
pentose-phosphate shunt (GO:0006098)	33	4	0.34	11.87	5.18E-04	5.00E-02
GO molecular function complete						
copper ion binding (GO:0005507)	191	10	1.95	5.13	4.11E-05	6.72E-02
hydrolase activity (GO:0016787)	2694	50	27.5	1.82	4.57E-05	4.98E-02
catalytic activity (GO:0003824)	8348	131	85.21	1.54	1.47E-08	4.80E-05
GO cellular component complete						
chloroplast inner membrane (GO:0009706)	63	5	0.64	7.77	6.30E-04	3.26E-02
plastid inner membrane (GO:0009528)	67	5	0.68	7.31	8.19E-04	3.84E-02
plastid stroma (GO:0009532)	751	31	7.67	4.04	1.07E-10	3.68E-08
chloroplast stroma (GO:0009570)	735	30	7.5	4	2.83E-10	7.30E-08
chloroplast thylakoid membrane (GO:0009535)	345	13	3.52	3.69	8.04E-05	5.19E-03
plastid thylakoid membrane (GO:0055035)	353	13	3.6	3.61	1.00E-04	5.75E-03
thylakoid membrane (GO:0042651)	381	14	3.89	3.6	5.57E-05	4.43E-03
photosynthetic membrane (GO:0034357)	386	14	3.94	3.55	6.37E-05	4.70E-03
plastid membrane (GO:0042170)	484	17	4.94	3.44	1.57E-05	1.35E-03
plastid envelope (GO:0009526)	861	30	8.79	3.41	9.64E-09	1.24E-06
cytosolic ribosome (GO:0022626)	294	10	3	3.33	1.13E-03	4.16E-02

chloroplast thylakoid (GO:0009534)	445	15	4.54	3.3	7.76E-05	5.35E-03
plastid thylakoid (GO:0031976)	451	15	4.6	3.26	8.96E-05	5.45E-03
chloroplast envelope (GO:0009941)	608	20	6.21	3.22	7.13E-06	7.36E-04
envelope (GO:0031975)	1283	38	13.1	2.9	6.70E-09	1.15E-06
organelle envelope (GO:0031967)	1283	38	13.1	2.9	6.70E-09	9.89E-07
thylakoid (GO:0009579)	547	15	5.58	2.69	6.56E-04	3.23E-02
plasmodesma (GO:0009506)	888	20	9.06	2.21	1.09E-03	4.88E-02
cell-cell junction (GO:0005911)	888	20	9.06	2.21	1.09E-03	4.67E-02
symplast (GO:0055044)	888	20	9.06	2.21	1.09E-03	4.49E-02
anchoring junction (GO:0070161)	888	20	9.06	2.21	1.09E-03	4.31E-02
cell junction (GO:0030054)	888	20	9.06	2.21	1.09E-03	4.15E-02
cytosol (GO:0005829)	2648	56	27.03	2.07	2.31E-07	2.66E-05
chloroplast (GO:0009507)	5068	105	51.73	2.03	1.12E-13	5.76E-11
plastid (GO:0009536)	5484	112	55.98	2	2.97E-14	3.07E-11
organelle membrane (GO:0031090)	1552	31	15.84	1.96	4.05E-04	2.20E-02
cytoplasm (GO:0005737)	14835	202	151.43	1.33	7.89E-10	1.63E-07
intracellular anatomical structure (GO:0005622)	21574	249	220.22	1.13	9.31E-06	8.75E-04

Analysis Type: PANTHER Overrepresentation Test (Released 20220712). Annotation Version and Release Date: GO Ontology database DOI: 10.5281/zenodo.6799722 Released 2022-07-01. Analyzed List: upload_1 (*Arabidopsis thaliana*). Reference List: *Arabidopsis thaliana* (all genes in database). Test Type: FISHER. Correction: FDR.

Supplementary Table S4. VQ1 interacting proteins with chloroplast localization identified in the Y2H screening.

AGI code	Symbol Uniprot	Annotation
At1g70760	NDHL	CRR23_NdhL__inorganic carbon transport protein-related
At3g04790	RPI3	Ribose 5-phosphate isomerase, type A protein
At5g66055	AKRP	AKRP_ ankyrin repeat protein
At2g32640	F4ITT3;Q0WVA1	LCYB Lycopene beta/epsilon cyclase protein
At5g24300	SSY1	ATSS1_ Starch synthase 1, chloroplastic/amyloplastic
AtCg00490	RBL	RBCL__ribulose-bisphosphate carboxylases
At5g53330	Q8LG11	Ubiquitin-associated/translation elongation factor EF1B protein
At1g79530	G3PP1	GAPCP-1__glyceraldehyde-3-phosphate dehydrogenase of plastid 1
At3g23640	Q93Y12/F4J460	HGL1__heteroglycan glucosidase 1/protein kinase-related
At3g06200	GMK3	Guanylate kinase 3, chloroplastic
At3g13390	Q9LJF2	L-ascorbate oxidase pectinesterase-like protein pollen-specific protein-like
At5g52320	Q9FHC8	CYP96A4__cytochrome P450, family 96, subfamily A, polypeptide 4
At1g64510	RR6	PRPS6__Translation elongation factor EF1B/ribosomal protein S6 family protein
At4g24460	CLT2	CLT2/CRT (chloroquine-resistance transporter)-like transporter 2
At3g59870	Q9M1Y7	Uncharacterized protein
At1g30510	FNRR2	ATRFNR2_ Ferredoxin--NADP reductase, root isozyme 2, chloroplastic
At5g14260	Q8VZB5	Rubisco methyltransferase family protein
At3g54050	F16P1	Fructose-1,6-bisphosphatase 1, chloroplastic/HCEF1__high cyclic electron flow 1
At5g19620	OEP80	ATOEP80_TOC75-V__outer envelope protein of 80 kDa
At5g04590	Q9LZ66;SIR	SIR__sulfite reductase
At3g54210	RK17	PRPL17__Ribosomal protein L17 family protein
At5g16390	BCCP1;F4KE21	BCCP_CAC1A__chloroplastic acetylcoenzyme A carboxylase 1
At5g65840	Q8L5Y8	Thioredoxin superfamily protein
At2g36250	FTZ21	ATFTSZ2-1_Tubulin/FtsZ family protein
At3g32930	Q9LHC4	6,7-dimethyl-8-ribityllumazine synthase
At2g33590	O22809	AtCRL1_NAD(P)-binding Rossmann-fold superfamily protein
At3g26900	SKL1	ATSKL1_shikimate kinase like 1
At5g50100	Y5010	DCC1__Putative thiol-disulphide oxidoreductase DCC
At2g34860	PSA2	PHOTOSYSTEM I ASSEMBLY 2, chloroplastic
At1g74070	CP26B	Cyclophilin-like peptidyl-prolyl cis-trans isomerase family protein

At1g56500	SOQ1	SUPPRESSOR OF QUENCHING 1, chloroplastic
At4g17560	RK191	50S Ribosomal protein L19-1
At1g19800	TGD1	ABCI14_TGD1__trigalactosyldiacylglycerol 1
At5g12040	NILP3	Omega-amidase, chloroplastic_Nitrilase/cyanide hydratase/N-acyltransferase
At4g15560	DXS	AtCLA1_DEF_DXPS2_DXS_DXS1__Deoxyxylulose-5-phosphate synthase
At1g74880	NDHO	NDH-O_NdhO__NAD(P)H:plastoquinone dehydrogenase complex subunit O
At3g01480	CYP38	Peptidyl-prolyl cis-trans isomerase_ATCYP38_ cyclophilin 38, chloroplastic
At4g32770	TOCC	VTE1/SDX1_tocopherol cyclase/ vitamin E deficient 1/ sucrose export defective 1
At4g32520	GLYP3	AtSHMT3_serine hydroxymethyltransferase 3
At1g34000	OHP2	Light-harvesting complex-like protein OHP2, chloroplastic/one-helix protein 2
At2g26340	F4IUI9;F4IUJ0	Uncharacterized protein
At2g30170	P2C26	PBCP_Protein phosphatase 2C protein 26
At1g75460	Q9FWT4	ATP-dependent protease La (LON) domain protein
At4g31990	B9DG21;F4JTH0	AAT3_ASP5_ATAAT1__aspartate aminotransferase 5
At1g76450	PPD3	Photosystem II reaction center PsbP domain-containing protein 3, chloroplastic_
At5g07020	MPH1	Maintenance of PSII under high light 1 MPH1 proline-rich family protein
At1g13270	F4HQQ8;MAP1B	MAP1B_methionine aminopeptidase 1B chloroplastic

Supplementary Table S5. Oligonucleotides used in this work.

Primer	Sequence (5'-3')	Loci	Application
VQ1-F	ATGTCTGCAGGAGTGAGATCTG	AT1G17147	Cloning *VQ1*
VQ1-R	TCAATGGTCTGACCAAAGATTATAC	AT1G17147	Cloning *VQ1*
VQ1nostop-R	ATGGTCTGACCAAAGATTATAC	AT1G17147	Cloning *VQ1*
VQ10-F	ATGTCTGGAAGAGGGAAAGTG	AT1G78410	Cloning *VQ10*
VQ10-R	TCAATATTCTGACCATAGTTTATACAATTC	AT1G78410	Cloning *VQ10*
VQ10nostop-R	ATATTCTGACCATAGTTTATA	AT1G78410	Cloning *VQ10*
T7	TAATACGACTCACTATAGGG	pGADT7-GW<br>pGBKT7-GW	Sequencing
M13-F	TGTAAAACGACGGCCAGT	pGADT7-GW<br>pGBKT7-GW<br>pCR8/GW/TOPO	Sequencing
M13-R	CAGGAAACAGCTATGACC	pCR8/GW/TOPO	Sequencing
35S-seq	CCTTCGCAAGACCCTTCCTCTA	pAlligator2	Sequencing
NOS-term	GCAAGACCGGCAACAGGATTCAATC	pAlligator2	Sequencing
attR1	GTTGTACAAAAAAGCAGGCT	pGWB454	Sequencing
5'pGADT7	CTATTCGATGATGAAGATACCCCACCAAACCC	pGADT7	Colony PCR and seq.
3'pGADT7	GTGAACTTGCGGGGTTTTTCAGTATCTACGATT	pGADT7	Colony PCR
